# Hydrochemical and Seasonally Conditioned Changes of Microbial Communities in the Tufa-Forming Freshwater Network Ecosystem

**DOI:** 10.1128/msphere.00602-22

**Published:** 2023-04-25

**Authors:** Andrea Čačković, Katarina Kajan, Lorena Selak, Tamara Marković, Andrijana Brozičević, Petra Pjevac, Sandi Orlić

**Affiliations:** a Division of Materials Chemistry, Ruđer Bošković Institute, Zagreb, Croatia; b Center of Excellence for Science and Technology-Integration of Mediterranean Region (STIM), Zagreb, Croatia; c Croatian Geological Survey, Zagreb, Croatia; d Scientific Research Center “Dr. Ivo Pevalek,” Plitvice Lakes National Park, Plitvička Jezera, Croatia; e Department of Microbiology and Ecosystem Science, Centre for Microbiology and Environmental Systems Science, University of Vienna, Vienna, Austria; f Joint Microbiome Facility of the Medical University of Vienna, Vienna, Austria; University of Michigan

**Keywords:** microbial communities, spatiotemporal, freshwater ecosystem, mass effects, species sorting, community assembly, tufa barriers

## Abstract

Freshwater network ecosystems consist of interconnected lotic and lentic environments within the same catchment area. Using Plitvice Lakes as an example, we studied the changes in environmental conditions and microbial communities (bacteria and fungi) that occur with downstream flow. Water samples from tributaries, interlake streams, connections of the cascading lakes, and the Korana River, the main outflow of the system, were characterized using amplicon sequencing of bacterial 16S rRNA and fungal ITS2 genes. Our results show that different environmental conditions and bacterial and fungal communities prevail among the three stream types within the freshwater network ecosystem during multiple sampling seasons. Microbial community differences were also confirmed along the longitudinal gradient between the most distant sampling sites. The higher impact of “mass effect” was evident during spring and winter, while “species sorting” and “environmental selection” was more pronounced during summer. Prokaryotic community assembly was majorly influenced by deterministic processes, while fungal community assembly was highly dominated by stochastic processes, more precisely by the undominated fraction, which is not dominated by any process. Despite the differences between stream types, the microbial community of Plitvice Lakes is shown to be very stable by the core microbiome that makes up the majority of stream communities. Our results suggest microbial community succession along the river-lake continuum of microbial communities in small freshwater network ecosystems with developed tufa barriers.

**IMPORTANCE** Plitvice Lakes represent a rare freshwater ecosystem consisting of a complex network of lakes and waterfalls connecting them, as well as rivers and streams supplying water to the lake basin. The unique geomorphological, hydrological, biogeochemical, and biological phenomenon of Plitvice Lakes lies in the biodynamic process of forming tufa barriers. In addition to microbial communities, abiotic water factors also have a major influence on the formation of tufa. Therefore, it is important to understand how changes in environmental conditions and microbial community assembly affect the functioning of the ecosystem of a freshwater network with developed tufa barriers.

## INTRODUCTION

Unique freshwater ecosystems consisting of lotic systems, such as streams and rivers, and lentic environments, such as lakes, connected within a catchment area, represent a spatial and temporal continuum from the source system to the mouth. Significant hydrologic and biogeochemical changes occur with community succession during runoff within lotic systems. Rivers and streams are primary receivers of nutrients and organic matter due to input from groundwater (1), soil, and surface runoff (2) but also from anthropogenic point sources (3). All this input with hydrology can significantly influence the microbial community in the ecosystem (4).

The streams’ water flow contributes to the structuring of microbial communities by massive advection of microbes from other systems through so-called “mass effects” (5, 6). Reaching lakes, microbial communities are massively influenced by the local hydrological and geochemical conditions, and through “environmental selection” and “species sorting,” the allochthonous communities can be displaced by more competitive species (7). Therefore, hydrology, the system position in a network and local environmental conditions, alongside community assembly processes (6) are the main influencers shaping freshwater microbial communities in network ecosystems.

Understanding the mechanisms underlying microbial community assembly, it is necessary to capture community composition across time and space. Temporal history shapes local communities, and unilateral water flow links temporal and spatial history. The control of the microbial community distribution patters is still an open box (8), but from the macroecological studies, we can identify four fundamental categories: selection, dispersal, diversification, and drift (9); the categories are shaped by the deterministic or stochastich microbial assembly processes (10). These processes are not mutually exclusive, and their relative importance alter the microbial diversity and its biological function (11).

Environmental filtering and interactions among species are regarded as deterministic selection processes (9, 12), whereas stochastic selection processes include random colonization, demographic coincidences, and ecological drift (13). The microbial community is formed as a net result of the upstream assembly processes due to the hydrological conditions in each of the subsystems within the aquatic network (14). In these networks, shifts in community compositions are not so much marked by the presence or absence of species but by changes in relative abundances (15). The community’s structure and thus the connection of the aquatic network are additionally shaped by seasonal hydrological fluctuations (16, 17). Thus, spatiotemporal studies are particularly important for a more detailed understanding of the functioning of freshwater network ecosystems. The main reason for the functioning of such network ecosystems is probably the existence of the core microbiome (15, 18, 19).

**FIG 1:**
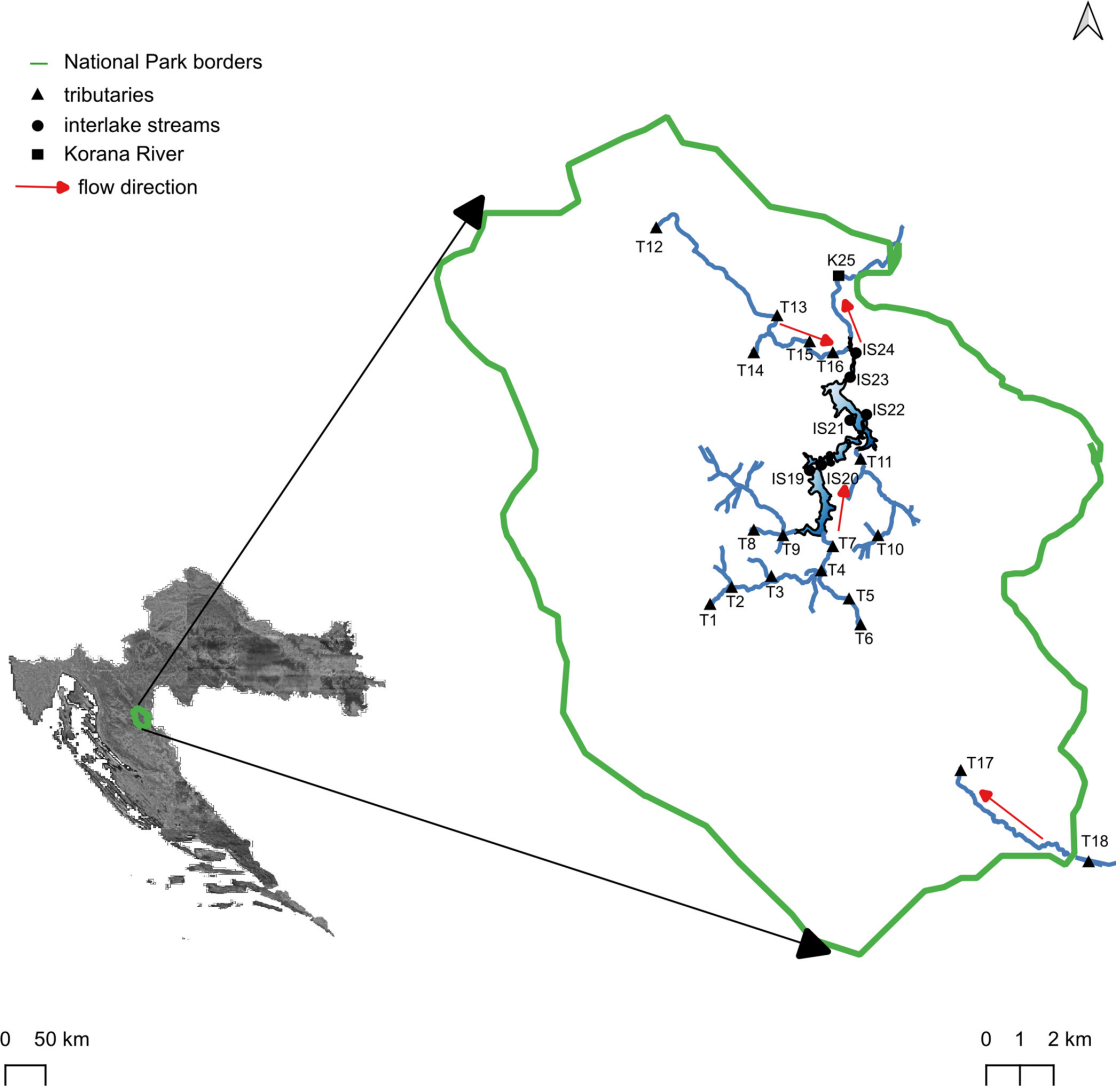
Locations of sampling points at streams in Plitvice Lakes catchment area. The map was generated using the software QGIS 3.24.

Plitvice Lakes represents a complex and biodiverse freshwater network ecosystem of lakes and waterfalls, as well as rivers and streams, providing the lake basin with water. Between the lakes are tufa barriers that form natural lake outlet habitats (20). In this complex environment (Fig. 1), we investigated the role of the spatial-temporal processes in the microbial community’s assembley and their response to different environmental conditions alongside the catchment area. Bacterial and fungal communities were examined throughout three different seasons during a period of 2 years. Our primary hypothesis was that there is a difference between microbial communities and assembley processes in tributaries, interlake streams, and the main outflow, the Korana River.

Considering the positions of streams in the network ecosystem, we expected the occurrence of the so-called mass effect downstream of the entire system. Similarly, the various microbial communities from forested tributaries were expected to undergo significant changes as they entered the lakes. These changes were expected to be due to species sorting, longer water residence time (WTR) in lakes, and different environmental conditions in the lakes. Finally, in order to better understand the functioning of the entire freshwater network ecosystem, the core microbiome was determined, which included taxa present in all stream types throughout all seasons.

## RESULTS

### Environmental variables.

All the physical and chemical parameters obtained at 25 sampling locations within the network ecosystem through different seasons are either shown in Fig. S1 or listed in Table S1. Water temperatures measured within tributaries were more stable and varied from 2°C (winter 2021) to 12°C (summer 2020) compared to interlake streams, which varied from 2°C (winter 2021) to 22°C (spring 2019; Fig. S1). The lowest measured temperature in the Korana River was 3°C (winter 2021), and the highest was 17°C (summer 2019). The lowest measured O_2_ concentration in tributaries was 4.8 mg/liter (summer 2019), and the highest was 12.9 mg/liter (winter 2021; Fig. S1). Similarly, in interlake streams and the Korana River, the lowest measured O_2_ concentrations were 6.9 and 4.7 mg/liter (summer 2019), and the highest were 12.6 (winter 2020) and 13.3 mg/liter (winter 2021). Measured dissolved organic (DOC) concentrations within all areas from spring 2019 to 2020 varied between 0.8 and 3.3 mg/liter (Fig. S1). The measured DOC concentration in summer 2020 in the Korana River was 6.9 mg/liter. The DOC concentrations peaked in winter 2021 in all streams, where they were up to 17.3 mg/liter in tributaries, 18.9 mg/liter in interlake streams, and 15.5 mg/liter in the Korana River, respectively. Generally, the lowest Ca^2+^ concentrations were measured in spring 2019 (Fig. S1). Highest Ca^2+^ concentrations in tributaries were measured in winter 2020 (89.4 mg/liter) and in interlake streams in winter 2021 (69.4 mg/liter). ${NO}_{3}^{-}$ concentrations were very low and varied between 0.6 mg/liter (in tributaries in winter 2020) and 6.8 mg/liter (in tributaries in winter 2021). One higher ${NO}_{3}^{-}$ concentration was measured within interlake streams in spring 2019 (14.9 mg/liter). The rainfall amounts differed in the same periods of the different years (Table S1). Spring samples were taken during the same period in May. However, it was rainy in May 2019, with a rainfall of 421.2 mm, while in May 2020, the rainfall was 161.5 mm. The summer samples in 2019 were taken in early September during dry season, with a rainfall of 52.3 mm, while the summer samples in 2020 were taken in the mid-September 2020, when rainfall already reached 217.8 mm. Winter samples were taken in February, which was a dry season in 2020 with a rainfall amount of 44.8 mm, while in 2021, it snowed during the same period, and the rainfall amount was 90.4 mm.

10.1128/msphere.00602-22.1 FIG S1 Environmental parameters measured at the sampling locations through six seasons. Download FIG S1, TIF file, 1.0 MB. Copyright © 2023 Čačković et al. 2023 Čačković et al. https://creativecommons.org/licenses/by/4.0/ This content is distributed under the terms of the Creative Commons Attribution 4.0 International license.

10.1128/msphere.00602-22.8 TABLE S1 Environmental parameters measured at 25 locations through 6 seasons. Download Table S1, DOCX file, 0.03 MB. Copyright © 2023 Čačković et al. 2023 Čačković et al. https://creativecommons.org/licenses/by/4.0/ This content is distributed under the terms of the Creative Commons Attribution 4.0 International license.

**FIG 2:**
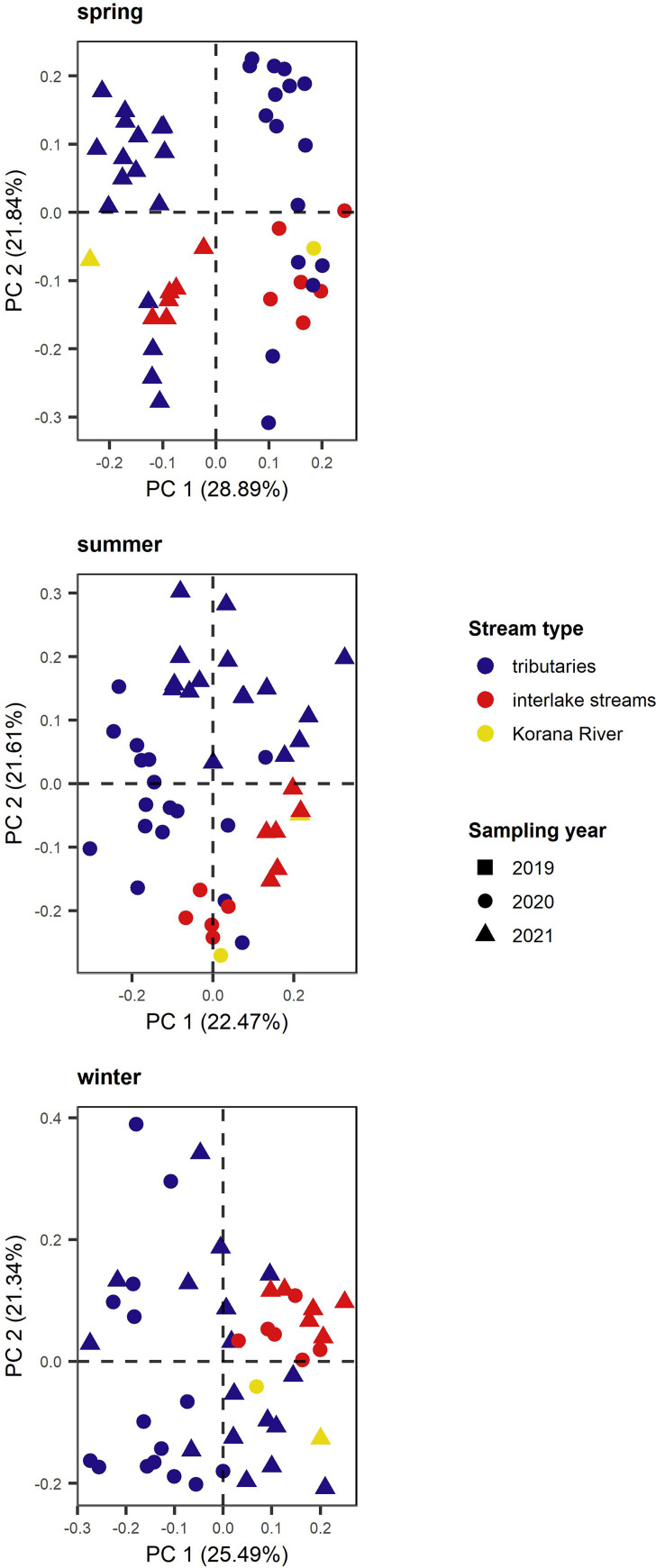
Principal component analysis (PCA) ordination on the environmental variables during the investigated period divided by sampling seasons. Different colors represent stream types.

Based on Pearson correlation coefficient temperature showed a negative correlation with DOC (*R^2^* = −0.39, *P < *0.05), O_2_ (*R^2^* = −0.38, *P < *0.05), and Ca^2+^ (*R^2^* = −0.45, *P < *0.05), while DOC and O_2_ (*R^2^* = 0.40, *P < *0.05) showed a positive correlation. Based on the principal component analysis (PCA) results, examined separately by season, the samples from spring and summer are divided on account of sampling year, while the samples from winter showed no clustering based on sampling year (Fig. 2). In the spring, samples from interlake streams were not separate from samples from tributaries, and in the summer and winter, little separation of samples from interlake streams and tributaries was evident. In general, interlake stream samples clustered together more than tributaries, which were scattered. The Korana River samples did not separate from the groups from the same years in the spring and were clustered with the interlake streams samples in the summer and the tributary samples in the winter.

### Microbial composition of freshwater network ecosystem.

α-Diversity of prokaryotic communities reached higher values in spring and winter in samples from tributaries than in samples from interlake stream or the Korana River, while values in summer were relatively the same for all stream types (Fig. S2A). Tukey’s honestly significant difference (HSD) test showed significant differences between tributaries and interlake streams and the Korana River in spring (*P < *0.05), whereas samples from interlake samples and the Korana River were not significantly different from each other in the same season (Table S2). For the winter samples, Tukey’s HSD test showed significant differences between all stream types, while no significant differences were detected for the summer samples (Table S2).

10.1128/msphere.00602-22.2 FIG S2 α-Diversity indices of prokaryotic (A) and fungal (B) communities determined by Pieolu Evenness, Chao1, and Shannon index. The samples are divided based on sampling season and then by stream type and color marked as follows: blue, tributaries; red, interlake streams; and yellow, Korana River. The sample abbreviations refer to different sampling locations. Download FIG S2, TIF file, 0.9 MB. Copyright © 2023 Čačković et al. 2023 Čačković et al. https://creativecommons.org/licenses/by/4.0/ This content is distributed under the terms of the Creative Commons Attribution 4.0 International license.

10.1128/msphere.00602-22.9 TABLE S2 Tukey’s honestly significant difference (HSD) test results. Download Table S2, DOCX file, 0.01 MB. Copyright © 2023 Čačković et al. 2023 Čačković et al. https://creativecommons.org/licenses/by/4.0/ This content is distributed under the terms of the Creative Commons Attribution 4.0 International license.

For the fungal communities, higher values for α-diversity were observed in the tributary samples in winter, while the values in spring and summer were similar to those observed in the interlake streams and Korana River samples (Fig. S2B). Tukey’s HSD test showed significant differences only among tributaries and interlake streams in winter (*P < *0.05; Table S2).

The taxonomic composition revealed, altogether, 54 prokaryotic (Fig. S3) and 12 different fungal phyla (Fig. S4). The most abundant bacterial phyla in all stream types were *Actinobacteriota*, *Bacteroidota*, *Cyanobacteria*, *Patescibacteria*, *Proteobacteria*, and *Verrucomicrobiota*. *Archaea* were overall rare. Among fungal phyla, *Ascomycota*, *Basidiomycota*, and *Chytridiomycota* were relatively most abundant.

10.1128/msphere.00602-22.3 FIG S3 Relative abundance of the total bacterial community structure (phylum level), divided by sampling seasons. The sample abbreviations refer to sampling locations. The “Other” group contains phyla with relative abundance of less than 1%. Download FIG S3, TIF file, 1.0 MB. Copyright © 2023 Čačković et al. 2023 Čačković et al. https://creativecommons.org/licenses/by/4.0/ This content is distributed under the terms of the Creative Commons Attribution 4.0 International license.

10.1128/msphere.00602-22.4 FIG S4 Relative abundance of the total fungal community structure (phylum level), divided by sampling seasons. The sample abbreviations refer to sampling locations. Download FIG S4, TIF file, 0.6 MB. Copyright © 2023 Čačković et al. 2023 Čačković et al. https://creativecommons.org/licenses/by/4.0/ This content is distributed under the terms of the Creative Commons Attribution 4.0 International license.

Bacterial amplicon sequence variants (ASVs) affiliated with *Actinobacteriota*, *Bacteroidota*, *Cyanobacteria*, and *Verrucomicrobiota* systematically increased in relative abundance from tributaries toward interlake streams (Fig. S3). Within tributaries, alongside the above-mentioned most abundant phyla, *Bdellovibrionota* appeared in higher relative abundances through all seasons. Higher relative abundances of *Cyanobacteria* in spring samples, *Firmicutes* and *Planctomycetota* in summer 2020, and *Campylobacterota* in winter 2021 were observed. In interlake streams, the relative abundances of *Bacteroidota* were lower in summer, while those of *Cyanobacteria* were higher. The Korana River samples showed similar bacterial community composition to interlake streams and followed the changes throughout the seasons (Fig. S3).

The fungal phylum *Chytridiomycota* dominated in the winter throughout almost all stream types, while *Ascomycota*- and *Basidiomycota*-related ASVs appeared in higher abundance in summer samples in all stream types (Fig. S4). In spring seasons, *Chytridiomycota* dominated in tributaries, while in spring 2020, *Ascomycota* and *Basidiomycota* dominated in interlake streams and the Korana River.

### Spatial-temporal shifts of microbial communities across the freshwater network ecosystem.

**FIG 3:**
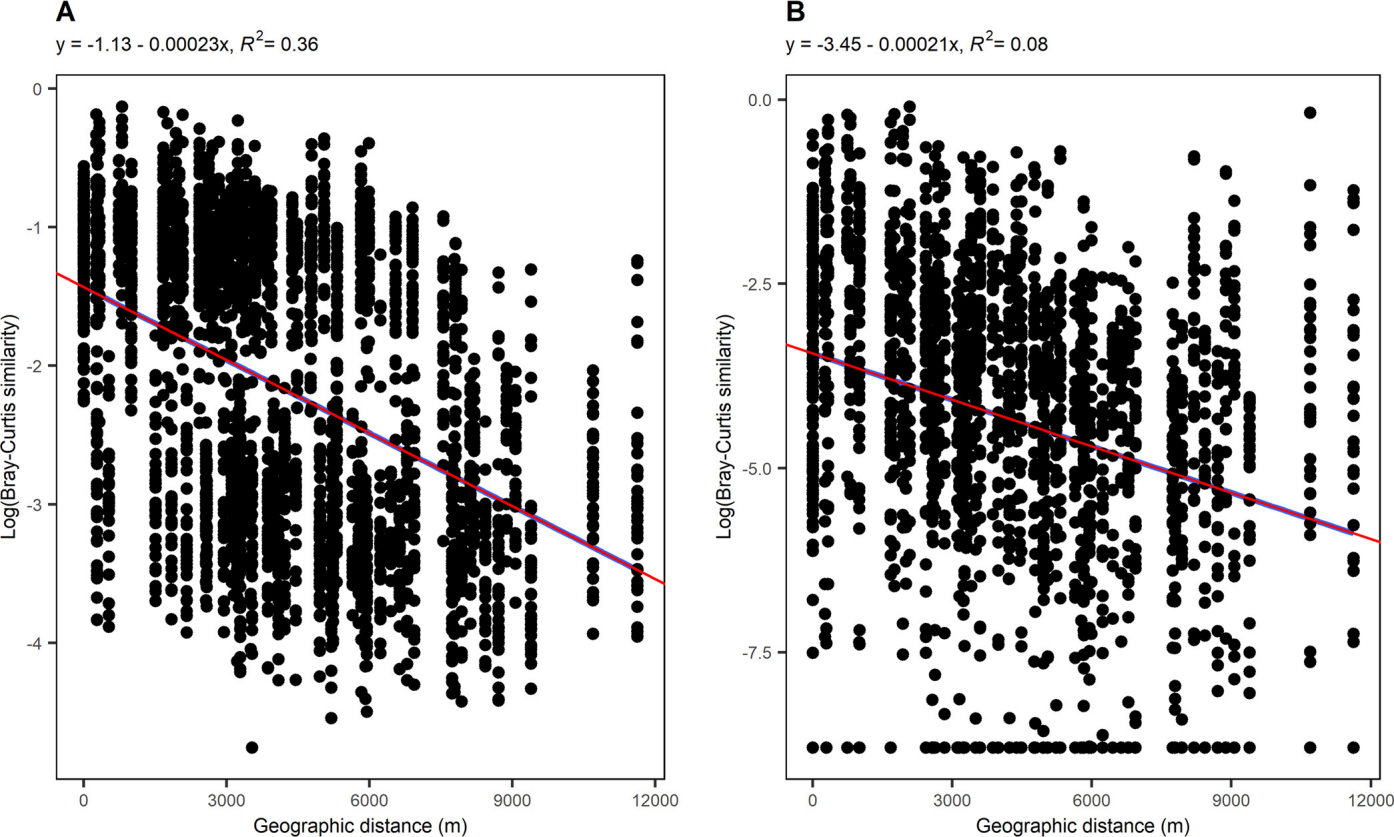
Principal coordinate analysis (PCoA) of bacterial (A) and fungal (B) communities’ β-diversity based on Bray-Curtis dissimilarity divided by sampling season. The influence of different stream type and sampling season on the sample clustering was tested by permutational multivariate analysis of variance (PERMANOVA). Different colors represent stream types, and different shapes represent sampling years.

Multivariate analysis revealed differences among microbial communities based primarily on the stream types, when all samples were examined across different sampling seasons. Clustering was also observed within the prokaryotic community in summer and winter between samples of the same stream type based on sampling year, while there was no difference between samples from different years in spring (Fig. 3A; Table S3). Prokaryotic communities of the Korana River clustered with the interlake stream communities. Within the fungal community, clustering between samples of the same stream type based on sampling year was most pronounced in winter, while in spring and summer, the influence of sampling year was significant but minor (Fig. 3B; Table S3). Fungal communities of the Korana River clustered with the communities of the tributaries in spring and winter, while in summer they clustered with the communities of interlake streams.

10.1128/msphere.00602-22.10 TABLE S3 Permutational multivariate analysis of variance (PERMANOVA) test results. Download Table S3, DOCX file, 0.01 MB. Copyright © 2023 Čačković et al. 2023 Čačković et al. https://creativecommons.org/licenses/by/4.0/ This content is distributed under the terms of the Creative Commons Attribution 4.0 International license.

### Main drivers of microbial community composition across the freshwater network ecosystem.

**FIG 4:**
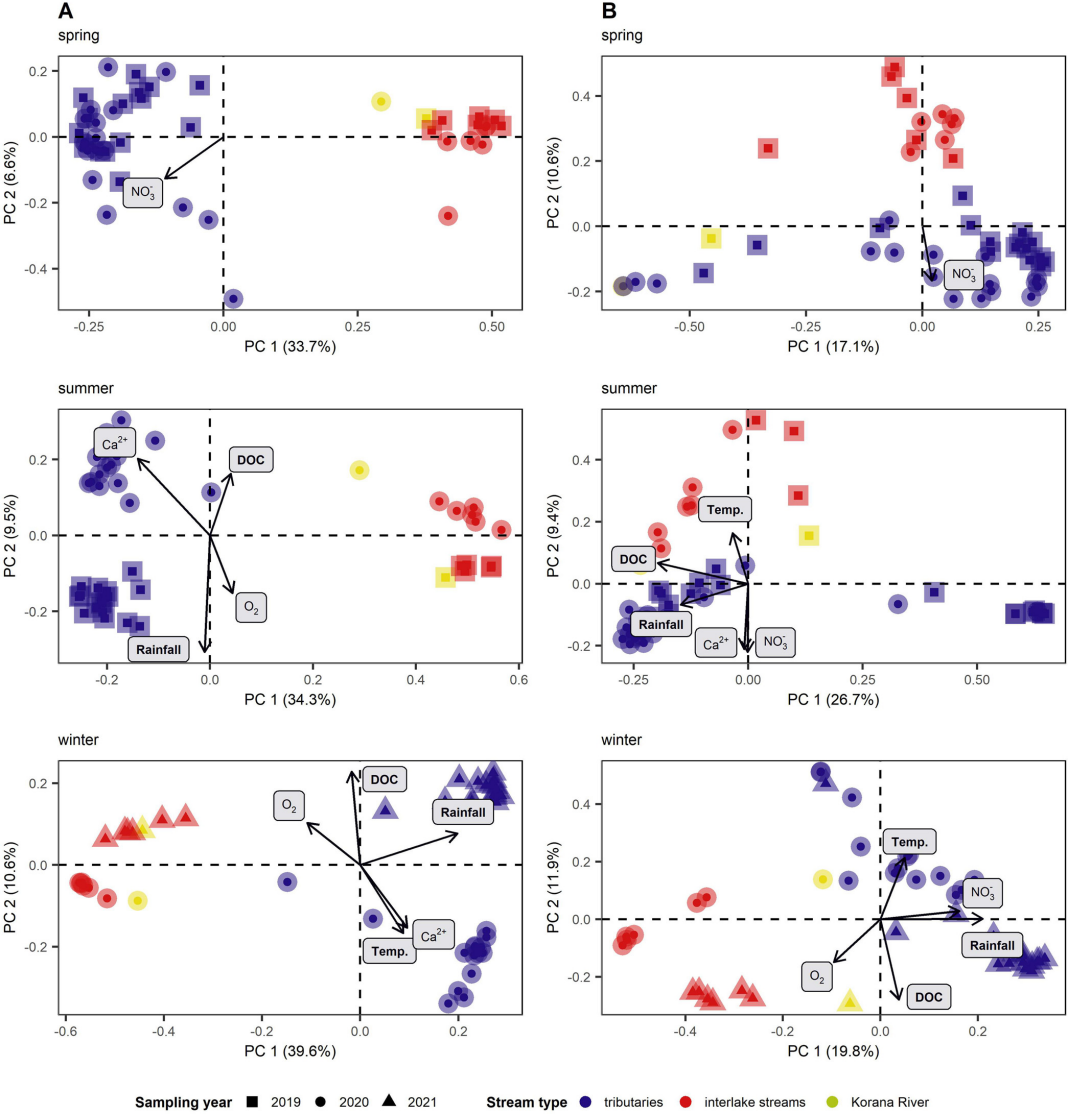
Pairwise Bray-Curtis community similarity between samples with respect to geographic distance (m). (A) Bacterial community. (B) Fungal community. For the evolution with distance, pairwise community similarity was evaluated exclusively between samples of the longest tributaries: Bijela Rijeka with interlake streams and Korana River. Blue lines illustrate linear models computed for the subset of samples considered, and red lines represent the overall linear regression when including all the samples.

To explore biogeographic differences of microbial communities along the longitudinal gradient, the community similarity was plotted as a function of geographic distance between sampling points (Fig. 4). The geographic distance between farthest sampling points was 12 km. Generally, microbial community similarity between samples decreased with increasing geographic distance (prokaryotic community slope, −0.00023; fungal community slope, −0.00021). Linear regression of the DDR showed a higher decrease in community similarity within the prokaryotic communities with increasing geographic distance (Fig. 4A), while the same observation was noticed for the fungal communities with slightly smaller values (Fig. 4B).

In order to discern the main drivers of the microbial distribution of the downstream flow within the freshwater ecosystem network depicted by the PCoA plots, the measured environmental variables were fitted on the separated ordinations (Fig. 3). Both prokaryotic and fungal community compositions were influenced by temperature, DOC, O_2_, Ca^2+^, ${NO}_{3}^{-}$, or rainfall. ${NO}_{3}^{-}$ influenced both communities of tributaries samples in spring. Tributary prokaryotic communities in summer were influenced by rainfall in 2019 and Ca^2+^ in 2020. In winter, temperature and Ca^2+^ influenced tributary prokaryotic communities in 2020, while rainfall amounts and DOC influenced tributary communities in 2021. Interlake stream prokaryotic communities were influenced by O_2_ in summer 2019, DOC in summer 2020, and O_2_ in winter 2021. Tributary fungal communities were influenced by ${NO}_{3}^{-}$ and Ca^2+^ and rainfall in summer, by temperature and ${NO}_{3}^{-}$ in winter 2020, and by rainfall and DOC in winter 2021. Fungal communities in interlake streams were influenced by temperature in summer 2020 and by O_2_ concentrations in winter 2021. The results were confirmed with permutational multivariate analysis of variance (PERMANOVA) (Table S3).

### Relative influence and quantitative analysis of assembly processes between different seasons.

**FIG 5:**
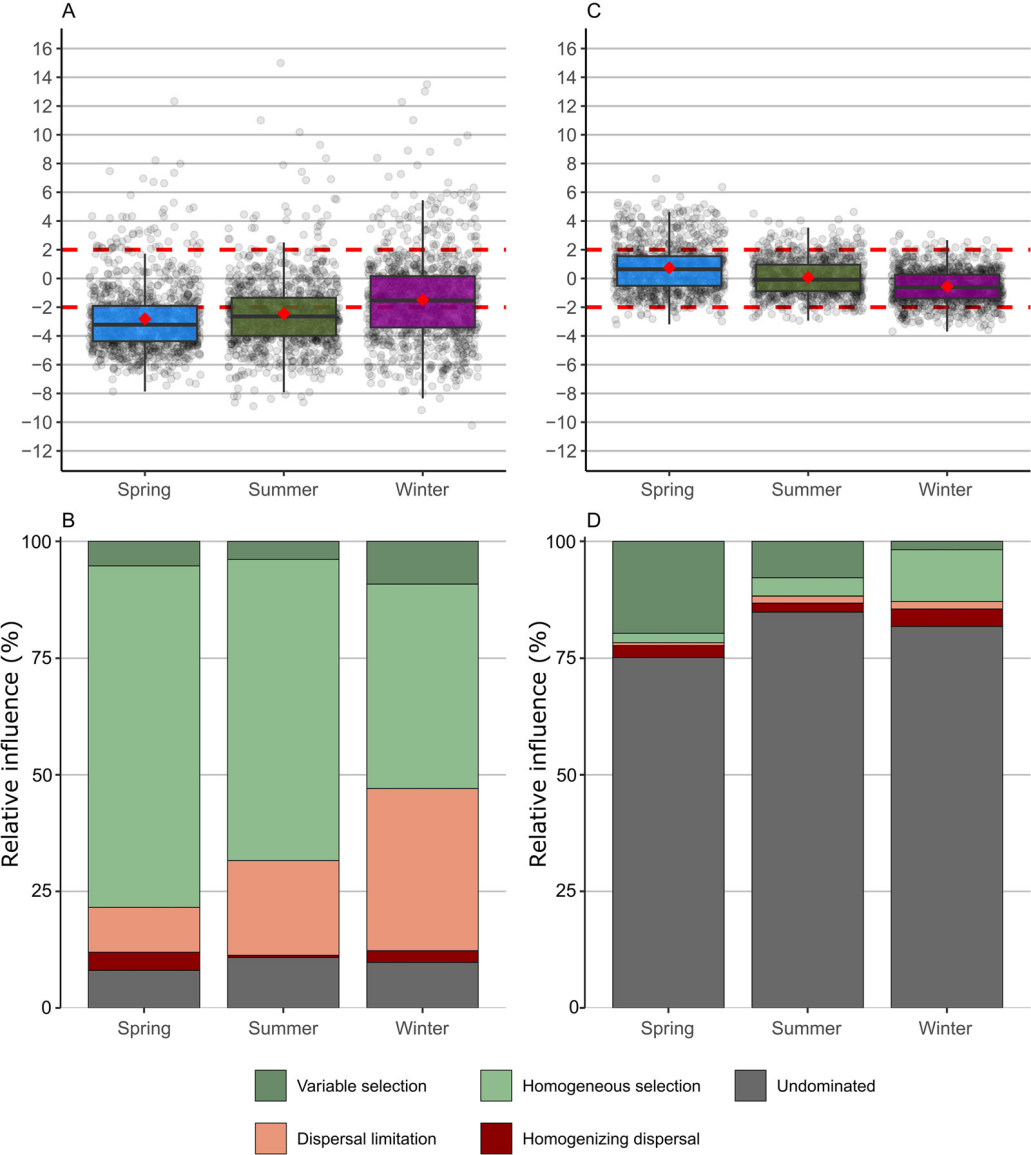
Community assembly processes for prokaryotic and fungal communities. Boxplots illustrating variation in β-nearest taxonomic index (βNTI) for prokaryotic (A) and fungal (C) communities from different seasons. The percentage of turnover in prokaryotic (B) and fungal (D) community assembly through different seasons governed primarily by various deterministic, including homogeneous and variable selection, and stochastic processes, including dispersal limitations and homogenizing dispersal, as well as the fraction that was not dominated by any single process (“Undominated”).

Partitioning the phylogenetic distance between prokaryotic ASVs using the null model, we found βNTI to be below −2 in spring and summer and below −2 in winter in some cases and above −2 in others, suggesting that stochastic assembly effected prokaryotic communities in winter (Fig. 5A). Homogeneous selection contributed to a large extent to the assembly of prokaryotic communities in all seasons (Fig. 5B). The highest contributions of homogeneous selection were found for prokaryotic communities in spring. Dispersal limitations also influenced prokaryotic community assembly in all seasons, with the greatest influence on prokaryotic communities in winter. Variable selection influenced prokaryotic communities more strongly in winter than in spring and summer. When we partitioned the phylogenetic distance between fungal ASVs using the null model, we also found that most βNTI ranged from −2 to +2 from site to site, suggesting that deterministic assembly had little influence on fungal community structuring (Fig. 5C). The processes that influenced fungal communities were mainly undominated. Homogeneous selection contributed to the assembly of fungal communities in spring, whereas variable selection influenced fungal communities in winter (Fig. 5D).

### Core microbiome of the freshwater network ecosystem.

**FIG 6:**
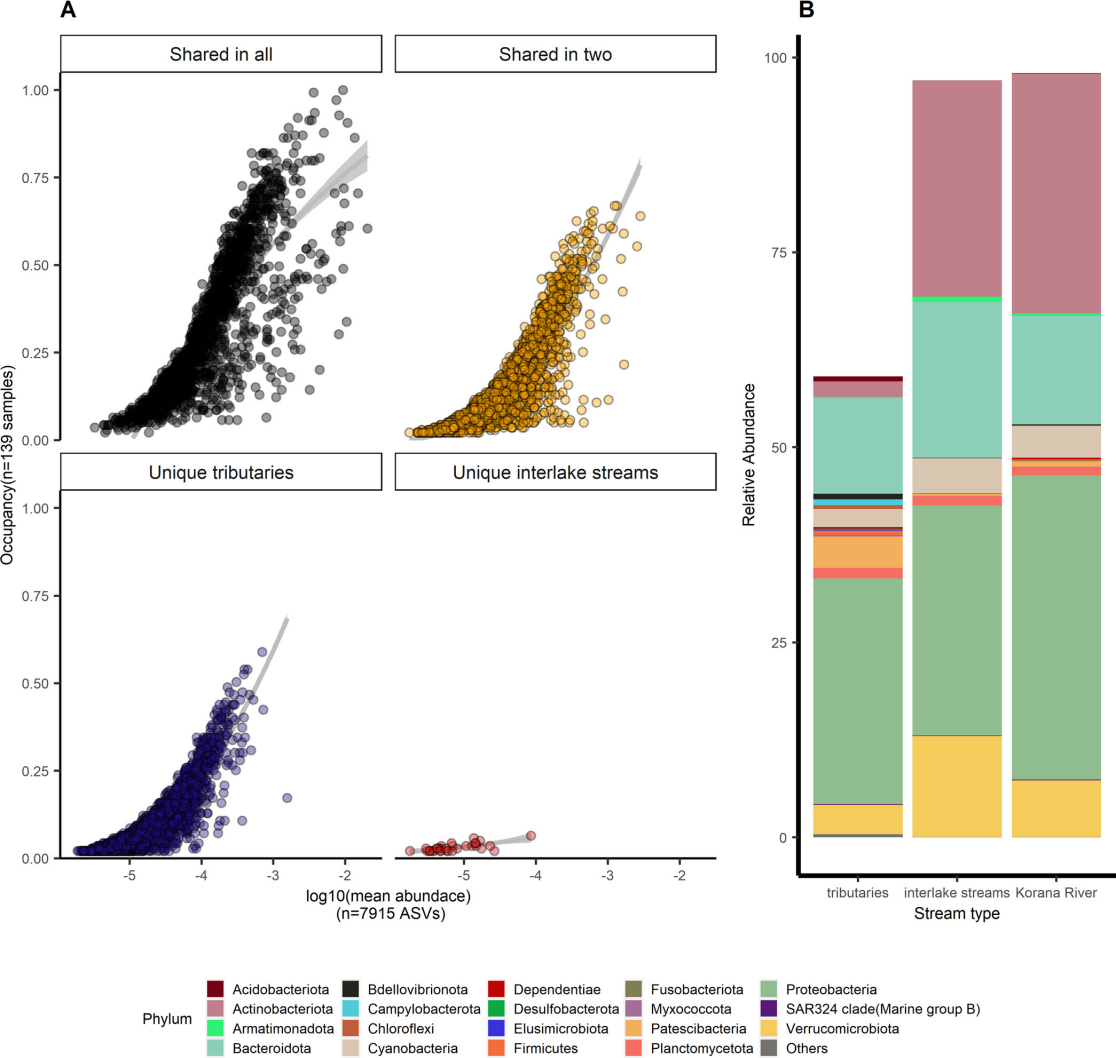
(A) Abundance-occupancy distributions were used to identify core members of the freshwater network ecosystem for bacteria. Taxa exclusive to stream types are indicated in blue (tributaries) or red (interlake streams), taxa shared between two stream types are indicated in yellow, and taxa shared across all systems are indicated in black. (B) Relative abundance of core microbiome taxa, grouped by stream type and color-coded by phylum. ASV, amplicon sequence variant.

**FIG 7:**
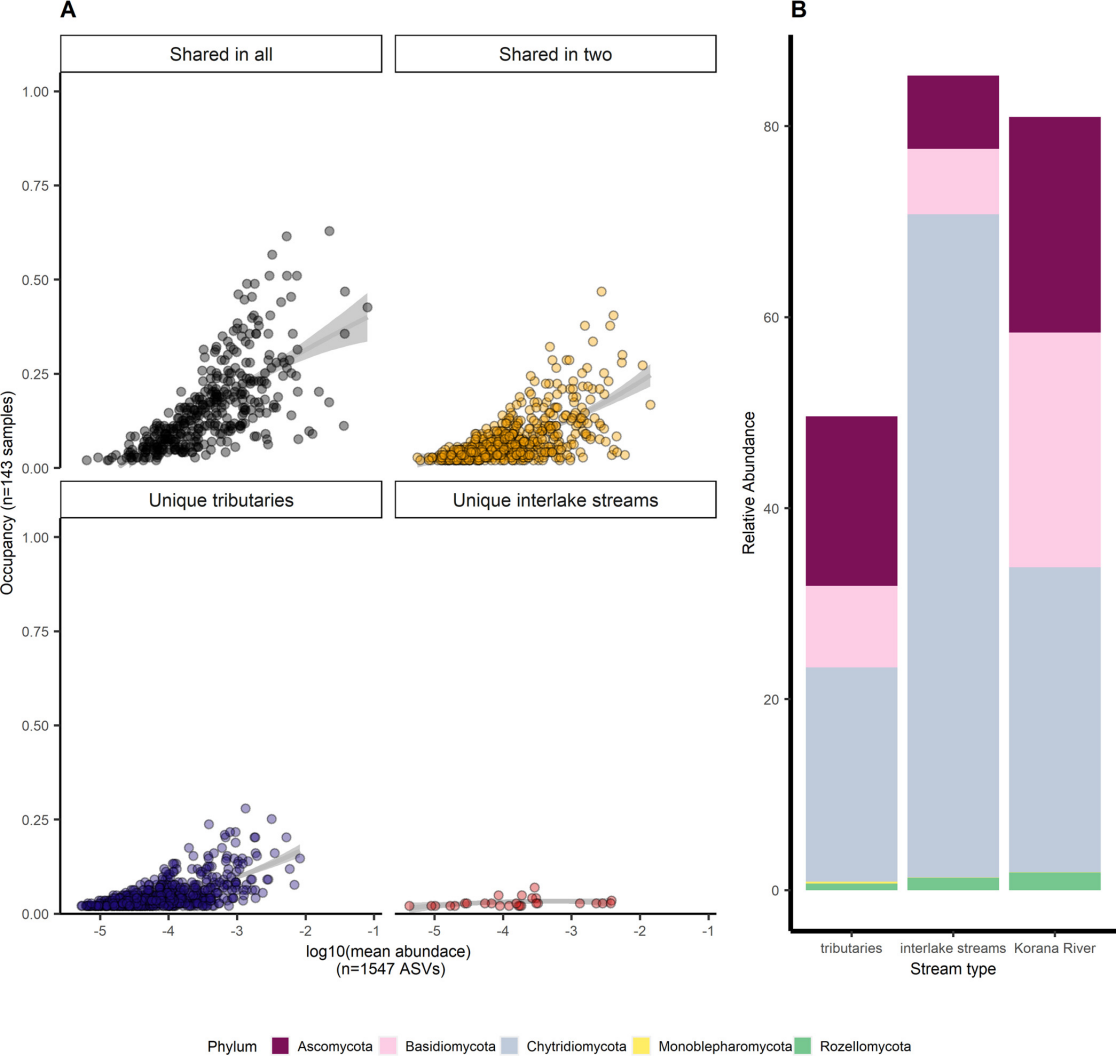
(A) Abundance-occupancy distributions were used to identify core members of the freshwater network ecosystem for fungi. Taxa exclusive to stream types are indicated in blue (tributaries) or red (interlake streams), taxa shared between two stream types are indicated in yellow, and taxa shared across all systems are indicated in black. (B) Relative abundance of core microbiome taxa, grouped by stream type and color-coded by phylum.

An analysis of the overlap between prokaryotic (Fig. S5A) and fungal (Fig. S5B) communities in the different sampling seasons showed that the majority of the taxa were unique to the tributaries. A considerable number of ASVs were shared by all three stream types or two stream types in all seasons. A minor number of ASVs was unique to interlake streams, while the Korana River had a maximum of 11 unique ASVs. The abundance-occupancy distributions of both bacterial (Fig. 6A) and fungal (Fig. 7A) communities showed the highest occupancy among samples of ASVs shared in all samples. Although the majority of ASVs were unique for tributaries, they were relatively rare. ASVs shared in all three stream types were also relatively most abundant in all samples and defined as core microbiome of this specific freshwater network ecosystem. The core microbiome covered 90% of the interlake stream and the Korana River and 70% of the tributary prokaryotic community (Fig. 6B). The most abundant ASVs were affiliated with the phyla *Proteobacteria*, *Bacteroidota*, and *Actinobacteria*. By exploring deeper taxonomic levels within the core microbiome, 195 different families have been discovered. In tributaries, bacteria from the *Comamonadaceae* family were most dominant, and bacteria from the *Flavobacteriaceae* and *Sphingomonadaceae* family were present in greater relative abundance than others. Downstream, i.e., in interlake streams and the Korana River, bacteria from the family *Sporichthyaceae* were dominant. Bacteria from the families *Clade III*, *Comamonadaceae*, *Microbacteriaceae*, and *Rubritaleaceae* were present in greater relative abundance (Fig. S6). In the fungal community, the core microbiome comprised 80% to –85% of interlake and Korana River communities and about 50% of tributary communities (Fig. 7B). The core microbiome of the fungal community was comprised of phyla *Ascomycota*, *Basidiomycota*, *Chytridiomycota*, *Monoblepharomycota*, and *Rozellomycota*. Among the ASVs unique for tributaries, *Proteobacteria*- and *Bacteroidota*-related ASVs in prokaryotic communities and *Chytridiomycota*-related ASVs in fungal communities prevailed. To better identify the uniqueness of tributaries, the 30 most abundant and rare families were investigated. The families *Chitinophagaceae*, *Leptolyngbyaceae*, *Saprospiraceae*, and *Spirosomaceae* were the most abundant, while *Erysipelatoclostridiaceae*, *Pelotomaculaceae*, and *Vibrionaceae* stood out among the rare taxa (Fig. S7). Although less numerous, ASVs shared in two stream types in bacterial communities were more abundant than unique ones and made 25% of bacterial and fungal communities of tributaries, while in interlake streams and the Korana River, ASVs made up less than 5% of bacterial and less than 20% of fungal communities.

10.1128/msphere.00602-22.5 FIG S5 Venn diagram representing the number of shared and unique bacterial (A) and fungal (B) ASVs between three different stream types: tributaries (green), interlake streams (red), and the Korana River (blue). Download FIG S5, TIF file, 0.8 MB. Copyright © 2023 Čačković et al. 2023 Čačković et al. https://creativecommons.org/licenses/by/4.0/ This content is distributed under the terms of the Creative Commons Attribution 4.0 International license.

10.1128/msphere.00602-22.6 FIG S6 Relative abundance of 30 most abundant core microbiome bacterial taxa, grouped by stream type and color-coded by family. Download FIG S6, TIF file, 0.5 MB. Copyright © 2023 Čačković et al. 2023 Čačković et al. https://creativecommons.org/licenses/by/4.0/ This content is distributed under the terms of the Creative Commons Attribution 4.0 International license.

10.1128/msphere.00602-22.7 FIG S7 Relative abundance of 30 most abundant and 30 most rare bacterial taxa unique for tributaries. The panels are color-coded by family. Download FIG S7, TIF file, 0.7 MB. Copyright © 2023 Čačković et al. 2023 Čačković et al. https://creativecommons.org/licenses/by/4.0/ This content is distributed under the terms of the Creative Commons Attribution 4.0 International license.

## DISCUSSION

In this study, we were primarily interested in determining changes in bacterial and fungal communities along the interconnected lotic systems during different seasons. Environmental parameters of the entire system were also determined during six sampling seasons.

In general, environmental parameters in samples from interlake streams showed overall uniformity, whereas the tributary samples were more scattered (Fig. 2). Accordingly, we expected differences in microbial communities among stream types, as previously shown in similarly studied aquatic network ecosystems (6, 21). Both bacterial and fungal communities (Fig. 3) showed differences in community composition between tributaries and downstream sites (interlake streams and the Korana River). Further confirmation of the different microbial communities in the tributaries and downstream can be provided by examining the biogeographic patterns of the microorganisms (Fig. 4). Indeed, when we examined the longest water flow in the Plitvice Lakes freshwater network ecosystem, it became clear that both bacterial and fungal communities differed with increasing geographic distance, that is, both microbial communities showed strong distance decay (21). The diversity of microbial communities was already evident at the level of phylum taxonomy, such that the strong dominance of phyla *Proteobacteria* and *Bacteroidota* in the tributaries was replaced by the sudden increase in the relative abundance of *Actinobacteriota* downstream in the network (22). Similarly, the dominance of *Chytridiomycota* was visible in the lower reaches, proving that rivers are dispersal pathways for terrestrial fungi (23–25), where fungi enter tributaries and flow downstream.

Based on environmental parameters, spring samples differed between sampling years but not between stream types (Fig. 2), while PCoA analysis indicated that there was a difference between microbial communities by stream type rather than sampling year (Fig. 3). Deterministically determined prokaryotic communities are mostly formed by the mass effect in spring (4). Namely, homogeneous selection influences community assembly when environmental conditions are relatively predictable (26). Under a low pressure of environmental changes (Table S3), bacterial communities that enter shallow tributaries from surrounding land, with water flow due to the strong mass effect, lose their abundance and diversity (6, 27) (Fig. S2). The only abiotic factor that affected bacterial community composition was the elevated ${NO}_{3}^{-}$ concentrations in tributaries (Fig. 3), which reach higher levels in karst aquifer systems during the wet season (28). For this very reason, we can conclude that species sorting and environmental selection had little effect on bacterial community formation. Fungal community assembly was largely stochastically influenced due to the undominated fraction (Fig. 5C and D). When we consider that most fungal ASVs were unclassified, this information is not surprising. Nonetheless, the small fraction of detected processes was dominated by variable selection, which causes high compositional turnover with large variations in environmental factors (26). Fungal communities were therefore more affected by environmental changes in spring (Table S3) but still mostly formatted by mass effect.

As might be expected, larger changes in environmental conditions occurred in the summer between the relatively sheltered, forested tributaries and the lower reaches of the system, which are subject to greater seasonal fluctuations (Fig. 2). The tributaries were obviously still strongly influenced by the surrounding soil, and the mass effect also shaped the entire community composition. However, depending on the environmental conditions during the summer, the influences of species sorting and environmental selection were stronger, especially in the lower reaches (4). Bacterial community assembly composition was still influenced by deterministic processes. Environmental factors had changed in terms of seasonality, but there have been no great leaps, so that the community composition was still influenced by homogeneous selection (26). Fungal community assembly was, again, largely stochastically influenced due to the undominated fraction (Fig. 5D).

In winter, samples from interlake streams were uniform according to environmental factors, whereas samples from tributaries were scattered (Fig. 2). A major impact on tributaries was a large jump in the concentration of DOC, which was elevated in 2021 due to snow (29). In winter, bacterial community assembly was dominated by deterministic processes, i.e., homogeneous selection. Nevertheless, the influence of stochastic processes was stronger here compared to other seasons (Fig. 5B). In winter, the movement of microorganisms to a new location is more limited; therefore, they showed a stronger dispersal limitation (8). The lesser influence of environmental factors (Table S3) and the differences in α- and β-diversity confirmed the greater impact of mass effect and more stochastic influence on bacterial community formation. The fungal communities were again not conditioned by any process (Fig. 5D), and homogeneous selection stood out from the detected processes. Changes in environmental parameters were not surprising, and therefore, communities were formed by homogeneous selection, but the parameters still had an effect on community changes (Table S3).

Overall, the Plitvice Lakes’ freshwater network ecosystem was characterized by relatively stable microbial communities with small seasonal changes due to communities present in all stream types, described as the core microbiome. Although the majority of bacterial ASVs were unique to the tributaries, they were rare and present in a smaller relative abundance (Fig. 6A), and the most significant part of the prokaryotic communities in tributaries was the core microbiome, also present in more than 90% of other stream types (Fig. 6B). *Proteobacteria*, already recognized earlier as transitional elements in a network ecosystem from streams to lakes (30), represented the most abundant phylum in the core microbiome. The phylum *Proteobacteria* was dominated by the chemotrophic *Comamonadaceae*, which are commonly found in groundwater and karst waters (reference 31 and references therein), primarily because of their role in utilizing various carbon sources (32). Other commonly occurring phyla were *Bacteroidota* and *Verrucomicrobiota*, which, together with *Proteobacteria*, represent the most profilic phyla in the bacterial communities of freshwater ecosystems (33). The most abundant families within the aforementioned phyla, *Flavobacteriaceae* (*Bacteroidota*) and *Rubritaleaceae* (*Verrucomicrobiota*), were bacteria normally associated with phytoplankton blooms and the degradation of polymeric matter (34). *Flavobacteriace* were more abundant in upstream samples because they are capable of degrading all organic matter present in the environment and are therefore important for biodegradation processes in subsurface karst environments (35). *Actinobacteriota* has already been associated with karst ecosystems (36), so it is not surprising that they are ubiquitous in Plitvice Lake’s network. Although they were present in low abundance in the tributaries, they experienced a sudden growth downstream, confirming their competitiveness (37). The most dominant family within the phylum *Actinobacteriota*, *Sporichthyaceae*, possesses phototrophic properties and is able to survive in oligotrophic environments (38). As photoheterotrophic bacteria, they use biodegradable DOC as a carbon source and produce their own DOC in combination with solar energy, which is why they dominated in downstream parts (38). Some unexpected phyla were present in the core microbiome, such as *Patescibacteria*, which are originally found in groundwater and aquifers, nutrient-poor habitats, where they make up as much as 20% of the microbial community (39, 40). Their high abundance in tributaries was not surprising as it is known that microbial communities are discharged with karst water to the surface (41). Still, their presence in lakes indicates their competitiveness and ecological roles in oxygen-rich environments (42). As for the core microbiome of the fungal community, a smaller percentage was present in all stream types (Fig. 7B) with a predominance of phylum *Chytridiomycota*, commonly found in aquatic habitats (43, 44) with various functionality from litter decomposition (45) to algae parasitism, when they potentially force cell death and cell wall disruption for organic matter release to decompose it directly (46). Representatives of the phyla *Ascomycota* and *Basidiomycota* were present in higher abundances in tributary and Korana River samples, probably due to their involvement in lichen symbioses (47). Also, the higher abundance of *Ascomycota* is associated with their important role in ecosystem succession (48). Dominance among taxa unique for tributaries in bacterial community was led by widespread forest soil bacteria from families *Chitinophagaceae* and *Saprospiraceae* (49) and *Leptolyngbyaceae*, which among other functions have the function of nitrogen fixation (50). Among rare taxa, bacteria from families associated with aromatic decomposition can be found, for instance *Pelotomaculaceae* (51). Taxa unique for tributaries and more diverse taxa shared in two stream types, constituted larger part of the fungal community (Fig. 7A). Various phyla, like *Mortierellomycota* and *Neocallimastigomycota*, showed a higher influence of soil and sediment on tributaries (44), same as *Olpidiomycota*, species that are typically internal parasites of algae, fungi, rotifers, and plant roots (52).

### Conclusions.

In summary, our study revealed the environmental and microbial differences between different stream types and sampling seasons within the freshwater network ecosystem. We have confirmed the stronger impact of soil on shallow tributaries, and we have shown that interlake streams show the condition of the lakes. Microbial communities in freshwater network ecosystems differed among stream types. In spring and winter, the mass effect had a greater influence on microbial community formation, while in summer, it was species sorting and environmental selection. Prokaryotic community assembly was influenced by deterministic processes throughout the system and in all seasons, while a lesser influence of stochastic processes was evident in winter. Fungal community assembly was dominated by stochastic processes. Despite minor changes in the communities through the seasons, we have shown that the microbial community of the Plitvice Lakes is very stable by determining the core microbiome. Still, due to the increasing anthropogenic influence and inevitable climate changes, it requires more profound research and monitoring.

## MATERIALS AND METHODS

### Study area.

The Plitvice Lakes National Park occupies 29.630,8 ha within the area of the Dinaric karst in west continental Croatia (Fig. 1). Less than 1% of this area is surface water. The aquatic ecosystem of the Plitvice Lakes consists of 16 cascading lakes, formed by the continuous biodynamic growth process of the tufa barriers that cut through this former river valley and enabled the formation of cascades.

Water samples were collected from all streams in the surface catchment area of Plitvice Lakes at 25 different locations (Fig. 1). The area of interest included three different stream types: tributaries (samples T1 to T18), interlake streams (samples IS19 to IS24), and the Korana River (sample K25). Tributaries included a total of 18 sampling locations, including several down the two leading water suppliers, the Bijela River, along its tributaries (Vukmirovića and Ljeskovac streams), and the Crna River. Tributaries also included locations along Sušanj and Rječica streams, which flow into the two largest lakes in the system, Prošćansko and Kozjak Lakes, as well as the more remote Korenička stream in Drakulić Rijeka and Rijeka Korenička. At last, three locations along the Plitvice stream and its affluent Sartuk stream were sampled. Together, they flow into the Korana River via the 76-m-high waterfall.

Interlake streams are the streams interconnecting the 16 lakes into a unique system. A total of six sampling locations were sampled at interlake streams: Labudovačke barriers, the exit barriers of Gradinsko Lake, a canal on Kozjački bridges, and the entrance barrier in Novakovića Brod Lake. Finally, a sample from the Korana River was taken as the central outflow of the entire Plitvice Lakes water system. Notably, the most distant tributaries are only 20 km separated from each other, and the geographic distance between the tributary of the longest flow, Bijela River, and the outflow of lakes, Korana River, is only 12 km.

At each sampling point, a total of 2 liters of water was collected in sterile polycarbonate (PC) bottles. Sampling was conducted during spring and summer in 2019 and 2020 and during winter in 2020 and 2021. The samplings in spring and winter were carried out in the same period both years; however, samplings in summer were done at the end of August 2019 and September 2020, respectively. The water samples were filtered onto a 0.22-μm-pore size PC filters (Whatman Nuclepore Track-Etch membrane; diameter, 47 mm) with a peristaltic pump. The filters were immediately stored at −20°C until DNA extraction, while the filtrates were preserved at 4°C for physicochemical analysis.

### Physicochemical analysis.

A Multisensor probe (EXO3, YSI, USA) was used to measure dissolved oxygen (O_2_), temperature, and pH *in situ*. Concentrations of the cations (Ca^2+^, Mg^2+^, and Na^+^) and anions (Cl^−^, ${SO}_{4}^{2-}$, ${NO}_{3}^{-}$) in filtered water samples were measured on a Dionex ICS-6000 DC (Thermo Fisher Scientific, Waltham, MA, USA). DOC and dissolved inorganic carbon (DIC) were analyzed using the HACH QBD1200 analyzer in filtered water samples. For DOC analysis, DIC was first removed (i.e., converted to CO_2_ and outgassed) by the addition of H_3_PO_4_. All analyses were performed in the Hydrochemical Laboratory of the Croatian Geological Survey.

### DNA extraction, amplification, and sequencing.

Total genomic DNA from filters was extracted with the DNeasy PowerWater kit (Qiagen, Inc., Valencia, CA, USA) following the manufacturer’s protocol. The hypervariable V4 region of the prokaryotic 16S rRNA gene was amplified by PCR using primer pair 515F Parada (5′-GTG YCA GCM GCC GCG GTA A-3′) (53) and 806R Apprill (5′-GGA CTA CNV GGG TWT CTA AT-3′) (54). The transcribed intergenic spacer 2 (ITS2) region of the fungal rRNA gene was amplified by PCR using primer pair ITS3-Mix1-Mix2 (TCCTCCGCTTAyTgATAtGc), a modified ITS3 Mix2 forward primer from Tedersoo48 named ITS3-mkmix2 CAWCGATGAAGAACGCAG, and a reverse primer ITS4 (equimolar mix of cwmix1 TCCTCCGCTTAyTgATAtGc and cwmix2 TCCTCCGCTTAtTrATAtGc) (44). As described in detail in reference 55, all samples were amplified, barcoded, purified, and prepared for sequencing on an Illumina MiSeq System (v3 chemistry, 2 × 300 bp) at the Joint Microbiome Facility of the Medical University of Vienna and the University of Vienna.

Individual amplicon pools were extracted from the raw sequencing data using the FASTQ workflow in BaseSpace (Illumina) with default parameters, allowing one mismatch for the 6-nucleotide (nt) library indexes. The input data were filtered for PhiX contamination with BBDuk (BBTools) (56). Further demultiplexing of each amplicon pool library into single amplicon libraries was performed with the python package demultiplex (Laros JFJ, github.com/jfjlaros/demultiplex), allowing one mismatch for barcodes and two mismatches for linkers and primer sequences, respectively.

ASVs were inferred using the DADA2 R package version 1.14.1 (57) with R version 3.6.1 (58) applying the recommended workflow (59) in pooled mode using all amplicon libraries per sequencing run. 16S rRNA region V4/V3-4 amplicon FASTQ reads were trimmed at 150/220 nt with allowed expected error 2. rRNA region ITS2 amplicon FASTQ reads were trimmed at 230/230 nt with allowed expected errors 4 and 6. Taxonomy was assigned to ASVs based on SILVA database SSU Ref NR 99 release 138.1 (https://www.ncbi.nlm.nih.gov/pubmed/23193283) and UNITE all eukaryotes general FASTA version 8.2 (60) using SINA version 1.6.1 (https://www.ncbi.nlm.nih.gov/pubmed/22556368).

The sequencing of V4 16S rRNA resulted in 1,187,393 reads, and the ITS sequencing resulted in a total of 359,819 reads. After filtering, the remaining 1,047,022 reads of the V4 16S rRNA gene clustered and affiliated with 7,915 prokaryotic ASVs and 316,582 reads of ITS2 fungal rRNA gene clustered and affiliated with 1,547 fungal ASVs were further analyzed. Due to a low number of reads, 7 samples were excluded from of the prokaryotic data set (sample T4_spring 2019, T2_summer 2019, T5_winter 2020, T18_ spring 2020, samples T14_summer 2020, and T16_summer2020, and T14_winter 2021), and further analysis was conducted on a total of 139 samples. A total of three samples (sample T4_spring 2019, IS19_summer 2019, and T14_winter 2021) were removed from the fungal data set because of a low number of reads. The analysis was conducted on 143 samples. In the prokaryotic data set, the lowest number of reads was determined within tributaries in spring 2020 (1,165 reads), and the highest was in interlake streams in summer 2019 (17,803 reads). The lowest number of reads within fungal data set was determined in tributaries in spring 2020 (105 reads), and the highest number was in interlake streams in winter 2021 (9,732 reads).

### Statistical analysis.

Statistical analyses were performed in the R environment (version 4.1.1) (58) using the packages phyloseq (61), vegan (62), dplyr (63), and ggplot2 (64). The β-diversity of environmental parameters was calculated by performing a PCA on a distance matrix of Z-score-normalized data using vegan.

Prior to statistical analysis, ASVs classified as eukaryotes, mitochondria, or chloroplasts in the 16S rRNA gene amplicon data set were removed. In addition, unassigned ASVs at the phylum level, singletons, and doubletons were removed from both data sets.

For estimation of α-diversity, rarefaction was computed on the data set by subsampling libraries to the smallest library size. α-Diversity was estimated as richness according to Chao1 (65), evenness was estimated according to reference 66, and diversity was estimated according to the Shannon index (67). The analysis of variance (ANOVA) test and Tukey’s HSD *post hoc* test were used to test for differences in microbial communities’ α-diversity among stream types. Taxonomic abundance was examined by removing unassigned taxa and by forming an “others” group of all the taxa with relative abundance less than 1%. Visualization of the distance decay relationship (DDR) relied on community similarity calculated using a Bray-Curtis index after normalization of the data set through cumulative sum scaling with the metagenomeSeq package (68). Geographic distance was measured using a “Vincenty” (ellipsoid) great circle distance to take into account Earth curvature, relying on packages enmSdm (69) and geosphere (v1.5.10) (70). The DDR was calculated between the samples belonging to the tributary with the longest flow, the Bijela River, all interlake stream samples, and the Korana River, the main outflow. Abundance-occupancy analysis was used to detect core taxa of entire freshwater network ecosystem (71). Each taxon’s mean relative abundance was calculated across the data set, log-transformed, and plotted against the proportion of discrete samples in which it occurred (with occupancy of 1 to be found in all samples). Taxa found in all sampling points were considered core taxa. Shared and unique ASVs of prokaryotic and fungal communities were depicted in a Venn diagram using the package ggVennDiagram (72). To capture the difference between prokaryotic and fungal β-diversities on a spatiotemporal scale between different stream types, sampling season, and sampling year, a PERMANOVA test was carried out on distance matrices based on Bray-Curtis dissimilarity and visualized via principal coordinate analysis (PCoA). The function envfit of the package vegan was applied to the results of PCoA to visualize the correlations with environmental factors.

A biodiversity ecological null model was used to evaluate processes driving microbial community composition (11, 73). Based on the rarified abundance ASV tables and amplicon phylogenetic trees, we calculated the β-nearest taxon index (βNTI) of prokaryotic and fungal communities. In order to test whether there was a significant difference between molecular and phylogenetic turnover between the observed microbial assemblages, the β-mean nearest taxon index (βMNTD) was calculated. Further, the βNTI was calculated as the difference between the observed βMNTD and the null distribution. Deterministic processes (variable or homogeneous selection) dominated when βNTI is greater than 2 or less than −2. Values within the range of 2 > NTI > −2 indicate the dominance of stochastic processes (homogenizing dispersal or dispersal limitation) or random drift. On the basis of the abundance of microbial communities, we calculated the Raup-Crick (RC) β-diversity to distinguish stochastic processes. Assemblies were structured by dispersal limitation if RC > +0.95, homogenizing dispersal if RC < −0.95, or random processes acting alone (ecological drift) if RC falls between −0.95 and +0.95.

### Data availability.

Raw sequence reads were deposited in the EBI-EMBL ENA database, project PRJEB57627.
