# Supplementary material for: Hydrochemical and Seasonally Conditioned Changes of Microbial Communities in the Tufa-Forming Freshwater Network Ecosystem
Source: mSphere. 2023 Apr 25;8(3):e00602-22. doi: 10.1128/msphere.00602-22 (PMC10291874; doi:10.1128/msphere.00602-22)
Supplement: TABLE S1 [file msphere.00602-22-s0001.docx]

| Sample | Stream type | Sampling season | pH | Turbidity | DIC (mg/L) | Mag^2+^ (mg/L) | SO_4_^2-^ (mg/L) | Na^+^ (mg/L) | Longitude | Latitude | Rainfall amounts (mm) |
| --- | --- | --- | --- | --- | --- | --- | --- | --- | --- | --- | --- |
| T1 | tributaries | spring 2019 | 7.70 | 0.34 | 12.46 | 27.71 | 2.88 | 1.38 | 44.83324 | 15.55742 | 421.22 |
| T2 | tributaries | spring 2019 | 8.45 | 0.89 | 10.93 | 20.42 | 0.96 | 0.46 | 44.84151 | 15.59712 | 421.22 |
| T3 | tributaries | spring 2019 | 8.46 | 0.95 | 14.24 | 28.93 | 3.84 | 0.69 | 44.83874 | 15.56471 | 421.22 |
| T4 | tributaries | spring 2019 | 8.37 | 1.08 | 11.26 | 26.25 | 0.96 | 0.92 | 44.84276 | 15.59948 | 421.22 |
| T5 | tributaries | spring 2019 | 8.37 | 1.08 | 11.26 | 26.25 | 0.96 | 0.92 | 44.828861 | 15.613972 | 421.22 |
| T6 | tributaries | spring 2019 | 7.94 | 0.34 | 10.75 | 17.50 | 2.88 | 0.69 | 44.8398 | 15.6 | 421.22 |
| T7 | tributaries | spring 2019 | 8.15 | 0.22 | 11.77 | 21.64 | 2.88 | 0.92 | 44.84855 | 15.60314 | 421.22 |
| T8 | tributaries | spring 2019 | 8.03 | 0.41 | 13.76 | 25.28 | 2.88 | 0.46 | 44.859792 | 15.578872 | 421.22 |
| T9 | tributaries | spring 2019 | 7.88 | 0.44 | 14.66 | 26.25 | 4.80 | 0.46 | 44.853382 | 15.589129 | 421.22 |
| T10 | tributaries | spring 2019 | 8.12 | 0.44 | 15.15 | 30.14 | 2.88 | 0.46 | 44.84929 | 15.62626 | 421.22 |
| T11 | tributaries | spring 2019 | 8.08 | 1.41 | 13.25 | 30.63 | 2.88 | 0.46 | 44.87457 | 15.61264 | 421.22 |
| T12 | tributaries | spring 2019 | 8.40 | 0.70 | 12.93 | 29.42 | 2.88 | 0.46 | 44.91136 | 15.57643 | 421.22 |
| T13 | tributaries | spring 2019 | 8.52 | 0.64 | 12.90 | 26.01 | 2.88 | 0.46 | 44.90627 | 15.57911 | 421.22 |
| T14 | tributaries | spring 2019 | 7.67 | 0.92 | 12.78 | 22.61 | 2.88 | 0.46 | 44.90148 | 15.57403 | 421.22 |
| T15 | tributaries | spring 2019 | 8.35 | 0.87 | 10.53 | 22.85 | 2.88 | 0.46 | 44.903833 | 15.593583 | 421.22 |
| T16 | tributaries | spring 2019 | 8.39 | 0.69 | 9.60 | 22.12 | 1.92 | 0.69 | 44.90234 | 15.60834 | 421.22 |
| T17 | tributaries | spring 2019 | 8.25 | 0.90 | 11.95 | 13.61 | 1.92 | 1.15 | 44.78385 | 15.65708 | 421.22 |
| T18 | tributaries | spring 2019 | 8.37 | 0.73 | 11.64 | 23.09 | 1.92 | 2.07 | 44.76938 | 15.69103 | 421.22 |
| IS19 | interlake streams | spring 2019 | 8.14 | 0.46 | 11.63 | 19.20 | 1.92 | 0.92 | 44.871571 | 15.596254 | 421.22 |
| IS20 | interlake streams | spring 2019 | 8.15 | 1.16 | 10.87 | 19.45 | 2.88 | 0.92 | 44.872169 | 15.599203 | 421.22 |
| IS21 | interlake streams | spring 2019 | 8.17 | 0.01 | 9.50 | 17.26 | 1.92 | 0.92 | 44.87945 | 15.61344 | 421.22 |
| IS22 | interlake streams | spring 2019 | 7.98 | 0.01 | 10.14 | 19.20 | 1.92 | 0.92 | 44.876972 | 15.613583 | 421.22 |
| IS23 | interlake streams | spring 2019 | 8.17 | 2.26 | 8.43 | 18.72 | 2.88 | 0.92 | 44.89443 | 15.60914 | 421.22 |
| IS24 | interlake streams | spring 2019 | 8.24 | 1.27 | 7.89 | 19.93 | 0.96 | 0.69 | 44.90189 | 15.6101 | 421.22 |
| K25 | Korana | spring 2019 | 8.43 | 0.41 | 7.83 | 19.93 | 1.92 | 0.69 | 44.924056 | 15.61475 | 421.22 |
| Sample | Stream type | Sampling season | pH | Turbidity | DIC (mg/L) | Mag^2+^ (mg/L) | SO_4_^2-^ (mg/L) | Na^+^ (mg/L) | Longitude | Latitude | Rainfall amounts (mm) |
| T1 | tributaries | summer 2019 | 7.44 | 0.54 | 7.60 | 24.80 | 2.88 | 1.38 | 44.83324 | 15.55742 | 52.30 |
| T2 | tributaries | summer 2019 | 8.14 | 4.72 | 6.31 | 20.42 | 1.92 | 0.46 | 44.84151 | 15.59712 | 52.30 |
| T3 | tributaries | summer 2019 | 8.24 | 1.12 | 7.41 | 29.90 | 3.84 | 0.46 | 44.83874 | 15.56471 | 52.30 |
| T4 | tributaries | summer 2019 | 8.10 | 1.67 | 6.42 | 20.42 | 2.88 | 0.92 | 44.84276 | 15.59948 | 52.30 |
| T5 | tributaries | summer 2019 | 7.41 | 0.40 | 3.72 | 18.23 | 3.84 | 0.92 | 44.828861 | 15.613972 | 52.30 |
| T6 | tributaries | summer 2019 | 8.04 | 0.34 | 1.22 | 20.18 | 4.80 | 0.69 | 44.8398 | 15.6 | 52.30 |
| T7 | tributaries | summer 2019 | 8.03 | 0.75 | 0.81 | 22.85 | 2.88 | 0.69 | 44.84855 | 15.60314 | 52.30 |
| T8 | tributaries | summer 2019 | 7.95 | 0.49 | 4.02 | 23.82 | 4.80 | 0.46 | 44.859792 | 15.578872 | 52.30 |
| T9 | tributaries | summer 2019 | 7.48 | 1.09 | 0.97 | 28.69 | 4.80 | 0.46 | 44.853382 | 15.589129 | 52.30 |
| T10 | tributaries | summer 2019 | 8.04 | 1.86 | 4.66 | 31.12 | 3.84 | 0.46 | 44.84929 | 15.62626 | 52.30 |
| T11 | tributaries | summer 2019 | 8.01 | 0.55 | 9.32 | 30.87 | 3.84 | 0.46 | 44.87457 | 15.61264 | 52.30 |
| T12 | tributaries | summer 2019 | 8.32 | 0.73 | 6.18 | 24.31 | 1.92 | 0.46 | 44.91136 | 15.57643 | 52.30 |
| T13 | tributaries | summer 2019 | 8.38 | 6.23 | 6.74 | 22.61 | 2.88 | 0.46 | 44.90627 | 15.57911 | 52.30 |
| T14 | tributaries | summer 2019 | 7.41 | 0.51 | 3.56 | 15.07 | 3.84 | 0.23 | 44.90148 | 15.57403 | 52.30 |
| T15 | tributaries | summer 2019 | 8.12 | 0.31 | 6.75 | 20.42 | 3.84 | 0.46 | 44.903833 | 15.593583 | 52.30 |
| T16 | tributaries | summer 2019 | 8.19 | 0.33 | 3.61 | 20.66 | 3.84 | 0.46 | 44.90234 | 15.60834 | 52.30 |
| T17 | tributaries | summer 2019 | 8.11 | 2.20 | 5.61 | 27.47 | 2.88 | 0.69 | 44.78385 | 15.65708 | 52.30 |
| T18 | tributaries | summer 2019 | 8.08 | 34.87 | 8.28 | 25.04 | 3.84 | 2.30 | 44.76938 | 15.69103 | 52.30 |
| IS19 | interlake streams | summer 2019 | 8.07 | 1.21 | 7.55 | 22.12 | 2.88 | 0.92 | 44.871571 | 15.596254 | 52.30 |
| IS20 | interlake streams | summer 2019 | 8.16 | 1.16 | 7.50 | 21.39 | 2.88 | 0.69 | 44.872169 | 15.599203 | 52.30 |
| IS21 | interlake streams | summer 2019 | 8.16 | 1.16 | 7.50 | 21.39 | 2.88 | 0.69 | 44.87945 | 15.61344 | 52.30 |
| IS22 | interlake streams | summer 2019 | 8.00 | 3.65 | 6.75 | 19.69 | 2.88 | 0.92 | 44.876972 | 15.613583 | 52.30 |
| IS23 | interlake streams | summer 2019 | 8.17 | 0.50 | 7.12 | 20.18 | 2.88 | 0.69 | 44.89443 | 15.60914 | 52.30 |
| IS24 | interlake streams | summer 2019 | 8.23 | 1.63 | 5.07 | 19.93 | 2.88 | 0.92 | 44.90189 | 15.6101 | 52.30 |
| K25 | Korana | summer 2019 | 8.17 | 0.31 | 0.74 | 19.20 | 1.92 | 0.69 | 44.924056 | 15.61475 | 52.30 |
| T1 | tributaries | winter 2020 | 7.80 | 1.72 | 15.32 | 30.48 | 3.84 | 1.61 | 44.83324 | 15.55742 | 44.80 |
| Sample | Stream type | Sampling season | pH | Turbidity | DIC (mg/L) | Mag^2+^ (mg/L) | SO_4_^2-^ (mg/L) | Na^+^ (mg/L) | Longitude | Latitude | Rainfall amounts (mm) |
| T2 | tributaries | winter 2020 | 8.31 | 0.65 | 14.84 | 22.12 | 2.88 | 0.69 | 44.84151 | 15.59712 | 44.80 |
| T3 | tributaries | winter 2020 | 8.30 | 0.42 | 16.57 | 34.96 | 4.80 | 0.69 | 44.83874 | 15.56471 | 44.80 |
| T4 | tributaries | winter 2020 | 8.31 | 0.65 | 14.84 | 22.12 | 2.88 | 0.69 | 44.84276 | 15.59948 | 44.80 |
| T5 | tributaries | winter 2020 | 7.52 | 0.38 | 14.44 | 16.82 | 3.84 | 1.15 | 44.828861 | 15.613972 | 44.80 |
| T6 | tributaries | winter 2020 | 8.16 | 0.35 | 14.28 | 18.48 | 3.84 | 1.15 | 44.8398 | 15.6 | 44.80 |
| T7 | tributaries | winter 2020 | 8.21 | 0.45 | 14.62 | 22.37 | 3.84 | 1.15 | 44.84855 | 15.60314 | 44.80 |
| T8 | tributaries | winter 2020 | 8.12 | 0.40 | 14.88 | 25.67 | 4.80 | 0.69 | 44.859792 | 15.578872 | 44.80 |
| T9 | tributaries | winter 2020 | 7.66 | 0.64 | 15.74 | 32.33 | 4.80 | 0.69 | 44.853382 | 15.589129 | 44.80 |
| T10 | tributaries | winter 2020 | 8.29 | 11.19 | 17.87 | 39.72 | 4.80 | 0.69 | 44.84929 | 15.62626 | 44.80 |
| T11 | tributaries | winter 2020 | 8.06 | 0.53 | 13.44 | 34.47 | 3.74 | 0.62 | 44.87457 | 15.61264 | 44.80 |
| T12 | tributaries | winter 2020 | 8.51 | 0.39 | 15.45 | 32.28 | 3.84 | 0.46 | 44.91136 | 15.57643 | 44.80 |
| T13 | tributaries | winter 2020 | 8.51 | 0.39 | 15.45 | 32.28 | 3.84 | 0.46 | 44.90627 | 15.57911 | 44.80 |
| T14 | tributaries | winter 2020 | 7.64 | 0.15 | 15.84 | 26.30 | 4.80 | 0.69 | 44.90148 | 15.57403 | 44.80 |
| T15 | tributaries | winter 2020 | 8.38 | 1.83 | 15.43 | 25.91 | 4.80 | 0.23 | 44.903833 | 15.593583 | 44.80 |
| T16 | tributaries | winter 2020 | 8.45 | 0.46 | 14.76 | 26.35 | 4.80 | 0.46 | 44.90234 | 15.60834 | 44.80 |
| T17 | tributaries | winter 2020 | 7.52 | 1.73 | 15.75 | 20.18 | 2.88 | 20.23 | 44.78385 | 15.65708 | 44.80 |
| T18 | tributaries | winter 2020 | 8.29 | 0.64 | 15.58 | 24.80 | 2.88 | 2.53 | 44.76938 | 15.69103 | 44.80 |
| IS19 | interlake streams | winter 2020 | 8.12 | 0.66 | 12.48 | 20.18 | 3.55 | 1.01 | 44.871571 | 15.596254 | 44.80 |
| IS20 | interlake streams | winter 2020 | 8.19 | 0.60 | 15.24 | 19.74 | 3.55 | 0.99 | 44.872169 | 15.599203 | 44.80 |
| IS21 | interlake streams | winter 2020 | 8.17 | 0.52 | 12.52 | 19.74 | 3.44 | 1.01 | 44.87945 | 15.61344 | 44.80 |
| IS22 | interlake streams | winter 2020 | 8.17 | 4.00 | 11.90 | 19.16 | 3.50 | 1.14 | 44.876972 | 15.613583 | 44.80 |
| IS23 | interlake streams | winter 2020 | 8.22 | 0.61 | 12.61 | 21.44 | 3.47 | 0.93 | 44.89443 | 15.60914 | 44.80 |
| IS24 | interlake streams | winter 2020 | 8.28 | 1.07 | 13.71 | 21.68 | 3.45 | 0.97 | 44.90189 | 15.6101 | 44.80 |
| IS25 | Korana | winter 2020 | 8.40 | 1.09 | 13.94 | 22.27 | 3.84 | 0.92 | 44.924056 | 15.61475 | 44.80 |
| T1 | tributaries | spring 2020 | 8.35 | 0.34 | 25.58 | 31.60 | 3.84 | 1.38 | 44.83324 | 15.55742 | 161.50 |
| T2 | tributaries | spring 2020 | 8.59 | 0.89 | 21.47 | 15.80 | 2.88 | 0.69 | 44.84151 | 15.59712 | 161.50 |
| Sample | Stream type | Sampling season | pH | Turbidity | DIC (mg/L) | Mag^2+^ (mg/L) | SO_4_^2-^ (mg/L) | Na^+^ (mg/L) | Longitude | Latitude | Rainfall amounts (mm) |
| T3 | tributaries | spring 2020 | 8.60 | 0.95 | 26.26 | 34.76 | 4.80 | 0.69 | 44.83874 | 15.56471 | 161.50 |
| T4 | tributaries | spring 2020 | 8.51 | 1.08 | 22.10 | 26.01 | 3.84 | 0.92 | 44.84276 | 15.59948 | 161.50 |
| T5 | tributaries | spring 2020 | 8.33 | 0.34 | 24.20 | 14.10 | 3.84 | 1.15 | 44.828861 | 15.613972 | 161.50 |
| T6 | tributaries | spring 2020 | 8.49 | 0.22 | 24.42 | 15.56 | 3.84 | 1.38 | 44.8398 | 15.6 | 161.50 |
| T7 | tributaries | spring 2020 | 8.53 | 0.41 | 24.75 | 19.20 | 3.84 | 0.92 | 44.84855 | 15.60314 | 161.50 |
| T8 | tributaries | spring 2020 | 8.40 | 0.44 | 25.63 | 27.71 | 4.80 | 0.69 | 44.859792 | 15.578872 | 161.50 |
| T9 | tributaries | spring 2020 | 8.37 | 0.46 | 25.89 | 29.42 | 4.80 | 0.69 | 44.853382 | 15.589129 | 161.50 |
| T10 | tributaries | spring 2020 | 8.45 | 0.44 | 22.90 | 33.55 | 3.84 | 0.46 | 44.84929 | 15.62626 | 161.50 |
| T11 | tributaries | spring 2020 | 8.23 | 0.30 | 25.85 | 34.76 | 3.84 | 0.69 | 44.87457 | 15.61264 | 161.50 |
| T12 | tributaries | spring 2020 | 8.60 | 0.70 | 21.94 | 30.87 | 2.88 | 0.69 | 44.91136 | 15.57643 | 161.50 |
| T13 | tributaries | spring 2020 | 8.72 | 0.64 | 22.17 | 29.90 | 2.88 | 0.69 | 44.90627 | 15.57911 | 161.50 |
| T14 | tributaries | spring 2020 | 8.37 | 0.92 | 24.55 | 19.69 | 3.84 | 0.69 | 44.90148 | 15.57403 | 161.50 |
| T15 | tributaries | spring 2020 | 8.47 | 0.87 | 24.61 | 20.18 | 3.84 | 0.69 | 44.903833 | 15.593583 | 161.50 |
| T16 | tributaries | spring 2020 | 8.51 | 0.69 | 24.31 | 20.66 | 4.80 | 0.69 | 44.90234 | 15.60834 | 161.50 |
| T17 | tributaries | spring 2020 | 8.36 | 0.90 | 21.69 | 13.86 | 2.88 | 0.69 | 44.78385 | 15.65708 | 161.50 |
| T18 | tributaries | spring 2020 | 8.48 | 0.73 | 22.48 | 23.34 | 2.88 | 2.30 | 44.76938 | 15.69103 | 161.50 |
| IS19 | interlake streams | spring 2020 | 8.35 | 0.31 | 23.98 | 19.93 | 3.84 | 0.92 | 44.871571 | 15.596254 | 161.50 |
| IS20 | interlake streams | spring 2020 | 8.36 | 0.36 | 24.36 | 19.93 | 3.84 | 0.92 | 44.872169 | 15.599203 | 161.50 |
| IS21 | interlake streams | spring 2020 | 8.41 | 0.13 | 23.77 | 19.20 | 3.84 | 0.92 | 44.87945 | 15.61344 | 161.50 |
| IS22 | interlake streams | spring 2020 | 8.43 | 0.26 | 23.76 | 19.45 | 2.88 | 0.92 | 44.876972 | 15.613583 | 161.50 |
| IS23 | interlake streams | spring 2020 | 8.41 | 0.57 | 23.60 | 21.15 | 2.88 | 0.92 | 44.89443 | 15.60914 | 161.50 |
| IS24 | interlake streams | spring 2020 | 8.49 | 0.69 | 23.75 | 21.64 | 3.84 | 0.92 | 44.90189 | 15.6101 | 161.50 |
| K25 | Korana | spring 2020 | 8.54 | 0.41 | 29.93 | 20.91 | 6.72 | 1.15 | 44.924056 | 15.61475 | 161.50 |
| T1 | tributaries | summer 2020 | 7.81 | 0.72 | 38.15 | 35.49 | 4.80 | 0.92 | 44.83324 | 15.55742 | 217.80 |
| T2 | tributaries | summer 2020 | 8.20 | 0.71 | 15.80 | 23.82 | 2.88 | 0.69 | 44.84151 | 15.59712 | 217.80 |
| T3 | tributaries | summer 2020 | 8.23 | 0.87 | 16.75 | 34.03 | 4.80 | 0.69 | 44.83874 | 15.56471 | 217.80 |
| Sample | Stream type | Sampling season | pH | Turbidity | DIC (mg/L) | Mag^2+^ (mg/L) | SO_4_^2-^ (mg/L) | Na^+^ (mg/L) | Longitude | Latitude | Rainfall amounts (mm) |
| T4 | tributaries | summer 2020 | 8.06 | 0.62 | 16.33 | 28.69 | 3.84 | 0.92 | 44.84276 | 15.59948 | 217.80 |
| T5 | tributaries | summer 2020 | 7.67 | 0.35 | 34.05 | 18.96 | 4.80 | 0.92 | 44.828861 | 15.613972 | 217.80 |
| T6 | tributaries | summer 2020 | 8.11 | 0.39 | 15.39 | 20.42 | 4.80 | 0.92 | 44.8398 | 15.6 | 217.80 |
| T7 | tributaries | summer 2020 | 7.95 | 2.16 | 15.77 | 22.85 | 2.88 | 0.92 | 44.84855 | 15.60314 | 217.80 |
| T8 | tributaries | summer 2020 | 7.73 | 0.30 | 36.67 | 29.17 | 3.84 | 0.69 | 44.859792 | 15.578872 | 217.80 |
| T9 | tributaries | summer 2020 | 7.85 | 0.73 | 16.58 | 31.60 | 3.84 | 0.69 | 44.853382 | 15.589129 | 217.80 |
| T10 | tributaries | summer 2020 | 8.13 | 1.03 | 16.71 | 35.49 | 2.88 | 0.46 | 44.84929 | 15.62626 | 217.80 |
| T11 | tributaries | summer 2020 | 8.12 | 0.38 | 16.86 | 36.22 | 3.84 | 0.69 | 44.87457 | 15.61264 | 217.80 |
| T12 | tributaries | summer 2020 | 8.46 | 0.60 | 16.47 | 33.06 | 2.88 | 0.69 | 44.91136 | 15.57643 | 217.80 |
| T13 | tributaries | summer 2020 | 8.52 | 0.64 | 16.07 | 32.82 | 2.88 | 0.69 | 44.90627 | 15.57911 | 217.80 |
| T14 | tributaries | summer 2020 | 7.80 | 0.50 | 36.53 | 23.82 | 3.84 | 0.46 | 44.90148 | 15.57403 | 217.80 |
| T15 | tributaries | summer 2020 | 8.23 | 0.55 | 15.89 | 25.28 | 3.84 | 0.46 | 44.903833 | 15.593583 | 217.80 |
| T16 | tributaries | summer 2020 | 8.29 | 0.42 | 21.83 | 25.04 | 3.84 | 0.46 | 44.90234 | 15.60834 | 217.80 |
| T17 | tributaries | summer 2020 | 8.19 | 1.00 | 16.38 | 27.96 | 2.88 | 0.69 | 44.78385 | 15.65708 | 217.80 |
| T18 | tributaries | summer 2020 | 8.16 | 0.84 | 16.24 | 27.96 | 3.84 | 2.53 | 44.76938 | 15.69103 | 217.80 |
| IS19 | interlake streams | summer 2020 | 8.16 | 0.44 | 14.58 | 22.61 | 2.88 | 0.92 | 44.871571 | 15.596254 | 217.80 |
| IS20 | interlake streams | summer 2020 | 8.21 | 0.40 | 14.55 | 21.88 | 2.88 | 0.92 | 44.872169 | 15.599203 | 217.80 |
| IS21 | interlake streams | summer 2020 | 8.29 | 0.23 | 13.85 | 20.18 | 2.88 | 0.92 | 44.87945 | 15.61344 | 217.80 |
| IS22 | interlake streams | summer 2020 | 8.26 | 0.53 | 14.00 | 19.93 | 2.88 | 0.92 | 44.876972 | 15.613583 | 217.80 |
| IS23 | interlake streams | summer 2020 | 8.27 | 0.39 | 14.22 | 22.85 | 3.84 | 0.92 | 44.89443 | 15.60914 | 217.80 |
| IS24 | interlake streams | summer 2020 | 8.38 | 0.24 | 13.69 | 22.85 | 3.84 | 0.92 | 44.90189 | 15.6101 | 217.80 |
| K25 | Korana | summer 2020 | 8.39 | 0.28 | 24.79 | 23.34 | 3.84 | 0.92 | 44.924056 | 15.61475 | 217.80 |
| T1 | tributaries | winter 2021 | 8.32 | 2.24 | 69.50 | 25.28 | 3.84 | 1.84 | 44.83324 | 15.55742 | 90.40 |
| T2 | tributaries | winter 2021 | 8.37 | 0.82 | 70.20 | 28.69 | 3.84 | 0.46 | 44.84151 | 15.59712 | 90.40 |
| T3 | tributaries | winter 2021 | 8.39 | 0.89 | 78.90 | 18.96 | 2.88 | 0.23 | 44.83874 | 15.56471 | 90.40 |
| T4 | tributaries | winter 2021 | 8.42 | 0.57 | 63.20 | 23.82 | 3.84 | 0.92 | 44.84276 | 15.59948 | 90.40 |
| Sample | Stream type | Sampling season | pH | Turbidity | DIC (mg/L) | Mag^2+^ (mg/L) | SO_4_^2-^ (mg/L) | Na^+^ (mg/L) | Longitude | Latitude | Rainfall amounts (mm) |
| T5 | tributaries | winter 2021 | 8.38 | 0.60 | 82.10 | 9.97 | 3.84 | 0.92 | 44.828861 | 15.613972 | 90.40 |
| T6 | tributaries | winter 2021 | 8.42 | 0.58 | 82.50 | 11.43 | 3.84 | 0.92 | 44.8398 | 15.6 | 90.40 |
| T7 | tributaries | winter 2021 | 8.35 | 1.01 | 56.00 | 15.56 | 2.88 | 0.92 | 44.84855 | 15.60314 | 90.40 |
| T8 | tributaries | winter 2021 | 8.39 | 0.69 | 43.50 | 23.58 | 2.88 | 0.23 | 44.859792 | 15.578872 | 90.40 |
| T9 | tributaries | winter 2021 | 8.30 | 0.72 | 69.70 | 25.53 | 4.80 | 0.46 | 44.853382 | 15.589129 | 90.40 |
| T10 | tributaries | winter 2021 | 8.51 | 0.88 | 60.90 | 29.90 | 3.84 | 0.23 | 44.84929 | 15.62626 | 90.40 |
| T11 | tributaries | winter 2021 | 8.00 | 0.71 | 60.90 | 29.90 | 3.84 | 0.23 | 44.87457 | 15.61264 | 90.40 |
| T12 | tributaries | winter 2021 | 8.27 | 0.79 | 66.50 | 26.25 | 3.84 | 1.61 | 44.91136 | 15.57643 | 90.40 |
| T13 | tributaries | winter 2021 | 8.20 | 0.83 | 66.80 | 26.25 | 2.88 | 0.23 | 44.90627 | 15.57911 | 90.40 |
| T14 | tributaries | winter 2021 | 8.05 | 0.55 | 69.90 | 17.99 | 3.84 | 0.23 | 44.90148 | 15.57403 | 90.40 |
| T15 | tributaries | winter 2021 | 8.04 | 0.66 | 61.70 | 18.72 | 3.84 | 0.23 | 44.903833 | 15.593583 | 90.40 |
| T16 | tributaries | winter 2021 | 8.01 | 0.72 | 69.70 | 18.48 | 3.84 | 0.23 | 44.90234 | 15.60834 | 90.40 |
| T17 | tributaries | winter 2021 | 8.44 | 0.76 | 78.20 | 8.99 | 3.84 | 1.38 | 44.78385 | 15.65708 | 90.40 |
| T18 | tributaries | winter 2021 | 8.50 | 1.04 | 85.30 | 17.50 | 3.84 | 2.76 | 44.76938 | 15.69103 | 90.40 |
| IS19 | interlake streams | winter 2021 | 8.34 | 0.63 | 61.70 | 14.59 | 3.84 | 0.92 | 44.871571 | 15.596254 | 90.40 |
| IS20 | interlake streams | winter 2021 | 8.32 | 0.71 | 60.90 | 14.59 | 3.84 | 0.92 | 44.872169 | 15.599203 | 90.40 |
| IS21 | interlake streams | winter 2021 | 8.26 | 0.56 | 63.90 | 15.07 | 3.84 | 0.92 | 44.87945 | 15.61344 | 90.40 |
| IS22 | interlake streams | winter 2021 | 8.28 | 8.56 | 62.90 | 14.83 | 3.84 | 0.92 | 44.876972 | 15.613583 | 90.40 |
| IS23 | interlake streams | winter 2021 | 8.02 | 0.65 | 60.90 | 17.26 | 2.88 | 0.69 | 44.89443 | 15.60914 | 90.40 |
| IS24 | interlake streams | winter 2021 | 8.53 | 0.57 | 55.50 | 17.26 | 2.88 | 0.69 | 44.90189 | 15.6101 | 90.40 |
| K25 | Korana | winter 2021 | 7.90 | 1.50 | 64.50 | 17.26 | 3.84 | 0.69 | 44.924056 | 15.61475 | 90.40 |
