# Supplementary material for: Hydrochemical and Seasonally Conditioned Changes of Microbial Communities in the Tufa-Forming Freshwater Network Ecosystem
Source: mSphere. 2023 Apr 25;8(3):e00602-22. doi: 10.1128/msphere.00602-22 (PMC10291874; doi:10.1128/msphere.00602-22)
Supplement: TABLE S2 [file msphere.00602-22-s0002.docx]

| BACTERIA |  |  |  |  |  | FUNGI |  |  |  |
| --- | --- | --- | --- | --- | --- | --- | --- | --- | --- |
| winter | | | | | | | | | |
|  | diff | lwr | upr | p.adj |  | diff | lwr | upr | p.adj |
| Chao1 ~ Stream type | | | | |  |  |  |  |  |
| tributaries-interlake streams | 1108,43 | 823,26 | 1393,6 | 1,58E-11 |  | 64,51 | 11,23 | 117,78 | 0,01 |
| tributaries-Korana | 992,73 | 378,69 | 1606,77 | 8,85E-04 |  | -69,83 | -184,9 | 45,25 | 0,31 |
| Korana-interlake streams | 115,70 | -527,74 | 759,14 | 9,01E-01 |  | 134,34 | 13,65 | 255,03 | 0,03 |
|  |  |  |  |  |  |  |  |  |  |
| Shannon ~ Stream type | | | | |  |  |  |  |  |
| tributaries-interlake streams | 2,19 | 1,52 | 2,86 | 1,60E-09 |  | 1,76 | 0,98 | 2,54 | 0,0000055 |
| tributaries-Korana | 2,46 | 1,02 | 3,9 | 4,44E-04 |  | 0,22 | -1,46 | 1,9 | 0,94 |
| Korana-interlake streams | -0,27 | -1,77 | 1,24 | 9,03E-01 |  | 1,54 | -0,23 | 3,3 | 0,098 |
|  |  |  |  |  |  |  |  |  |  |
| Evenness ~ Stream type | | | | |  |  |  |  |  |
| tributaries-interlake streams | 701,89 | 541,59 | 862,18 | 1,50E-12 |  | 38,01 | 14,83 | 61,18 | 0,00074 |
| tributaries-Korana | 677,47 | 332,31 | 1022,62 | 6,40E-05 |  | 5,09 | -44,98 | 55,16 | 0,97 |
| Korana-interlake streams | 24,47 | -337,26 | 386,1 | 9,90E-01 |  | 32,92 | -19,6 | 85,43 | 0,29 |
|  |  |  |  |  |  |  |  |  |  |
| summer | | | | | | | | | |
|  | diff | lwr | upr | p.adj |  | diff | lwr | upr | p.adj |
| Chao1 ~ Stream type | | | | |  |  |  |  |  |
| tributaries-interlake streams | 227,84 | -207,57 | 663,25 | 0,42 |  | -8,21 | -62,9 | 46,49 | 0,93 |
| tributaries-Korana | 173,16 | -764,38 | 1110,7 | 0,9 |  | 6,06 | -105,1 | 117,22 | 0,99 |
| Korana-interlake streams | 54,68 | -927,75 | 1037,1 | 0,98 |  | -14,27 | -132,79 | 104,26 | 0,95 |
|  |  |  |  |  |  |  |  |  |  |
| Shannon ~ Stream type | | | | |  |  |  |  |  |
| tributaries-interlake streams | -0,02 | -0,95 | 0,91 | 0,99 |  | -0,49 | -1,56 | 0,58 | 0,51 |
| tributaries-Korana | 0,32 | -1,68 | 2,33 | 0,92 |  | -0,53 | -2,71 | 1,65 | 0,83 |
| Korana-interlake streams | -0,34 | -2,45 | 1,76 | 0,92 |  | 0,04 | -2,28 | 2,36 | 0,99 |
|  |  |  |  |  |  |  |  |  |  |
| Evenness ~ Stream type | | | | |  |  |  |  |  |
| tributaries-interlake streams | 42,92 | -189,79 | 275,62 | 0,9 |  | -10,92 | -33,92 | 12,09 | 0,49 |
| tributaries-Korana | 81 | -420,06 | 582,06 | 0,92 |  | -7,42 | -54,17 | 39,34 | 0,92 |
| Korana-interlake streams | -38,08 | -563,13 | 486,97 | 0,98 |  | -3,5 | -53,35 | 46,35 | 0,98 |
|  |  |  |  |  |  |  |  |  |  |
| spring | | | | | | | | | |
|  | diff | lwr | upr | p.adj |  | diff | lwr | upr | p.adj |
| Chao1 ~ Stream type | | | | |  |  |  |  |  |
| tributaries-interlake streams | 1009,64 | 731,81 | 1287,47 | 8,5E-11 |  | 8,7 | -27,74 | 45,14 | 0,83 |
| tributaries-Korana | 648,42 | 48,24 | 1248,6 | 0,032 |  | 58,76 | -20,2 | 137,72 | 0,18 |
| Korana-interlake streams | 361,22 | -268,26 | 990,69 | 0,35 |  | -50,06 | -132,94 | 32,83 | 0,32 |
|  |  |  |  |  |  |  |  |  |  |
| Shannon ~ Stream type | | | | |  |  |  |  |  |
| tributaries-interlake streams | 1,8 | 1,1 | 2,5 | 0,00000047 |  | 0,0077 | -0,69 | 0,71 | 0,99 |
| tributaries-Korana | 1,46 | -0,06 | 2,97 | 0,062 |  | 1,24 | -0,27 | 2,76 | 0,13 |
| Korana-interlake streams | 0,34 | -1,25 | 1,93 | 0,86 |  | -1,24 | -2,83 | 0,36 | 0,16 |
|  |  |  |  |  |  |  |  |  |  |
| Evenness ~ Stream type | | | | |  |  |  |  |  |
| tributaries-interlake streams | 431,78 | 332,57 | 530,99 | 1,2E-12 |  | 0,91 | -11,25 | 13,06 | 0,98 |
| tributaries-Korana | 351,53 | 137,2 | 565,86 | 0,00073 |  | 24,82 | -1,51 | 51,16 | 0,07 |
| Korana-interlake streams | 80,25 | -144,54 | 305,04 | 0,66 |  | -23,92 | -51,56 | 3,73 | 0,1 |
