# Supplementary material for: Hydrochemical and Seasonally Conditioned Changes of Microbial Communities in the Tufa-Forming Freshwater Network Ecosystem
Source: mSphere. 2023 Apr 25;8(3):e00602-22. doi: 10.1128/msphere.00602-22 (PMC10291874; doi:10.1128/msphere.00602-22)
Supplement: TABLE S3 [file msphere.00602-22-s0003.docx]

|  | BACTERA |  |  |  |  |  | FUNGI |  |  |  |  |  |
| --- | --- | --- | --- | --- | --- | --- | --- | --- | --- | --- | --- | --- |
|  | SPRING |  | SUMMER |  | WINTER |  | SPRING |  | SUMMER |  | WINTER |  |
|  | *r^2^* | *p* | *r^2^* | *p* | *r^2^* | *p* | *r^2^* | *p* | *r^2^* | *p* | *r^2^* | *p* |
| Temperature | - | - | 0.99109 | 0.032 * | 0.9704 | 0.011 * | 0.08227 | 0.001 *** | 0.0857 | 0.001 *** | 0.07619 | 0.001 *** |
| DO | - | - | - | - | - | - | 0.04893 | 0.003 ** | 0.09285 | 0.001 *** | 0.06513 | 0.001 *** |
| DOC | - | - | 0.96748 | 0.004 ** | - | - | - | - | 0.05966 | 0.003 ** | 0.08091 | 0.001 *** |
| Calcium | - | - | 0.81651 | 0.003 ** | - | - | 0.06444 | 0.001 *** | 0.06046 | 0.003 ** | 0.04931 | 0.008 ** |
| Nitrate | 0.42781 | 0.001 *** | 0.34443 | 0.001 *** | 0.48377 | 0.002 ** | - | - | 0.04013 | 0.034 * | 0.0583 | 0.004 ** |
| Rainfall |  |  |  |  |  |  | 0.06133 | 0.001 *** | 0.09656 | 0.001 *** | 0.11534 | 0.001 *** |
|  |  |  |  |  |  |  |  |  |  |  |  |  |
| Stream type | 0.33458 | 0.001 *** | 0.35179 | 0.001 *** | 0.38557 | 0.001 *** | 0.13862 | 0.001 *** | 0.10637 | 0.001 *** | 0.19477 | 0.001 *** |
| Sampling year | - | - | 0.08528 | 0.002 ** | 0.10051 | 0.002 ** | 0.06133 | 0.001 *** | 0.09656 | 0.001 *** | 0.10127 | 0.001 *** |
